# Similar Transcriptomic Responses to Early and Late Drought Stresses Produce Divergent Phenotypes in Sunflower (*Helianthus annuus* L.)

**DOI:** 10.3390/ijms24119351

**Published:** 2023-05-27

**Authors:** Garrett M. Janzen, Emily L. Dittmar, Nicolas B. Langlade, Nicolas Blanchet, Lisa A. Donovan, Andries A. Temme, John M. Burke

**Affiliations:** 1 Department of Plant Biology, University of Georgia, Athens, GA 30602, USA; 2 INRA Centre de Toulouse Midi-Pyrénées, 31320 Castanet-Tolosan, France; 3 LIPME, Université de Toulouse, INRAE, CNRS, 31320 Castanet-Tolosan, France

**Keywords:** sunflower, drought, stress, compensation, overcompensation, gene expression, gene co-expression

## Abstract

Cultivated sunflower (*Helianthus annuus* L.) exhibits numerous phenotypic and transcriptomic responses to drought. However, the ways in which these responses vary with differences in drought timing and severity are insufficiently understood. We used phenotypic and transcriptomic data to evaluate the response of sunflower to drought scenarios of different timing and severity in a common garden experiment. Using a semi-automated outdoor high-throughput phenotyping platform, we grew six oilseed sunflower lines under control and drought conditions. Our results reveal that similar transcriptomic responses can have disparate phenotypic effects when triggered at different developmental time points. Leaf transcriptomic responses, however, share similarities despite timing and severity differences (e.g., 523 differentially expressed genes (DEGs) were shared across all treatments), though increased severity elicited greater differences in expression, particularly during vegetative growth. Across treatments, DEGs were highly enriched for genes related to photosynthesis and plastid maintenance. A co-expression analysis identified a single module (M8) enriched in all drought stress treatments. Genes related to drought, temperature, proline biosynthesis, and other stress responses were overrepresented in this module. In contrast to transcriptomic responses, phenotypic responses were largely divergent between early and late drought. Early-stressed sunflowers responded to drought with reduced overall growth, but became highly water-acquisitive during recovery irrigation, resulting in overcompensation (higher aboveground biomass and leaf area) and a greater overall shift in phenotypic correlations, whereas late-stressed sunflowers were smaller and more water use-efficient. Taken together, these results suggest that drought stress at an earlier growth stage elicits a change in development that enables greater uptake and transpiration of water during recovery, resulting in higher growth rates despite similar initial transcriptomic responses.

## 1. Introduction

Drought is an environmental stress of global agronomic concern [1,2]. Global climate change is already affecting drought frequency in several geographic regions [3,4] and the severity and variability in drought frequency and duration is projected to further increase [5,6], thereby threatening food security. There is thus considerable interest in understanding how plants respond to drought stress as we work to develop more resilient crops.

The concept of plant drought resistance strategies (escape, avoidance, tolerance) comes from the classic literature of Levitt [7], Turner [8], and Ludlow and Muchow [9]. It is based on the observation that “responses to water stress and associated characteristics do not occur at random among plants. Rather they are grouped in combinations called strategies” [7]. First, drought escape involves rapid development such that plants have either reproduced or gone dormant before water limitation impacts them. Second, drought avoidance is associated with the ability to maintain plant water status closer to an unstressed level via traits that maximize water uptake and/or minimize water loss. Such traits include lower stomatal conductance ($g_{s}$), higher water-use efficiency (WUE), deeper rooting, and altered growth to increase the root mass ratio [10,11]. Finally, drought tolerance is usually associated with the ability to physiologically tolerate low plant water status by lowering the turgor loss point via osmotic adjustment [12]. These strategies and trait responses are not mutually exclusive, but rather form a continuum.

While there is a large body of work on drought resistance mechanisms, key questions relating to how variations in drought timing and severity impact plant drought response remain unanswered. Indeed, plant responses to drought during early development may differ from responses to stress during flowering [13,14]. In addition to timing, the severity of drought stress may influence the nature of the response [15], and responses that promote drought resistance under moderate stress can be deleterious under severe stress, and vice versa [16]. Moreover, lingering effects of drought on plant functions during a recovery period in which water availability increases remain poorly understood. From a molecular perspective, the genes involved in the drought response itself vs. drought recovery likely differ [17]. For example, Liu et al. [18] found that genes/pathways may be variably up/downregulated between the dehydration and rehydration phases in *Camellia*. Similarly, in maize, drought-tolerant lines demonstrated greater overall differential gene expression during recovery than during drought itself, suggesting that recovery strategies can play a central role in overall drought resilience [19]. Though differential expression takes place in the hours and days after rehydration [17], some responses are specific to certain time windows [20] and the extent to which longer-term transcriptomic changes persist through later development remains largely unexplored.

Here we investigate the phenotypic and transcriptomic responses of cultivated sunflower (*Helianthus annuus* L.) to drought scenarios that vary in both timing and severity. Sunflower, which is one of the world’s most important oilseed crops and is frequently grown on rain-fed lands, is generally recognized as a drought resistant crop due to its ability to both avoid and tolerate water limitation [21]. Avoidance is largely achieved through a large, “explorative” root system that enhances moisture uptake during water deficit [22], while tolerance is thought to be achieved via osmotic adjustment [23,24] as well as changes in growth. For example, leaf area is generally reduced under drought, which limits transpirational water loss [22,24,25,26,27], but at the expense of photosynthesis [17]. Despite its general drought-hardiness due to these and other aspects of its biology, drought stress consistently ranks as one of the primary factors limiting sunflower production [28]. While some breeding efforts have focused on adapting sunflowers to drier conditions [29,30], much work remains to be carried out on this front.

A better understanding of the mechanisms underlying drought responses will inform efforts aimed at developing more resilient crops. In our work, we sought to disentangle the effects of timing and severity on both the phenotypic and transcriptomic responses of sunflower to drought, and to further investigate plant growth/performance as well as transcriptional changes during the post-drought recovery period. To this end, we conducted a water limitation experiment using the state-of-the-art semi-automated outdoor high-throughput phenotyping platform Heliaphen [31] that enables a controlled drydown of plants to specific levels of water availability. We were thus able to induce multiple controlled drought scenarios in which soil moisture was consistently maintained at target soil water deficits regardless of factors such as plant size (and thus rates of water uptake). We used this platform, combined with the automated collection of phenotypic data and RNA-Seq analyses, to answer the following questions: (1) does sunflower exhibit different phenotypic and/or transcriptomic responses to drought conditions implemented at different severities and/or developmental stages?; (2) how does the timing of drought (i.e., early drought followed by recovery vs. a late, terminal drought) affect plant performance?; and (3) do the phenotypic and/or transcriptomic effects of drought persist when plants are allowed to recover after an early vegetative stress?

## 2. Results

### 2.1. Phenotypic Analyses

#### 2.1.1. Phenotypic Means and Variance

**Figure 1:**
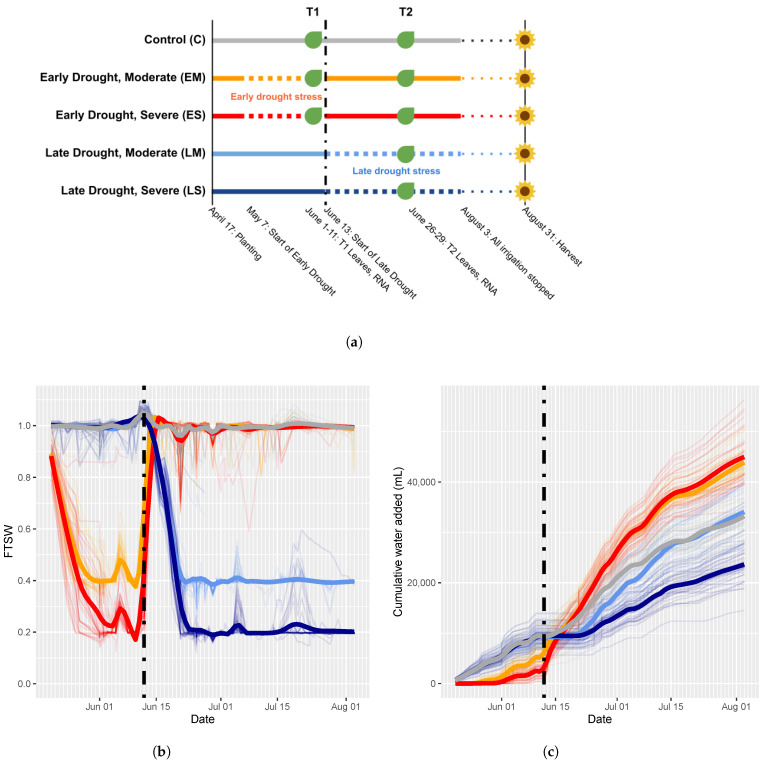
Drought treatment implementation. (**a**) Experimental design. Irrigation treatments control (C), early moderate (EM), early severe (ES), late moderate (LM), and late severe (LS) were applied to five groups of plants. Vertical dashed line represents the date on which the early drought conditions ended and the late drought conditions began. RNA was sampled at two time points (T1 and T2) during vegetative and reproductive developmental phases. At T1, LM and LS treatments were identical to the control treatment, so RNA was not sampled for those treatments at that time point. This design resulted in the following eight treatment/timing groups: C_T1_, EM_T1_, ES_T1_, C_T2_, EM_T2_, ES_T2_, LM_T2_, LS_T2_. (**b**) Fraction of transpirable soil water (FTSW) tracks the soil water moisture levels over the course of the experiment. (**c**) Mean total water provided to each treatment group over the course of the experiment.

Plants were grown under one of five irrigation treatment groups: control (C), early moderate drought stress (EM), early severe drought stress (ES), late moderate drought stress (LM), and late severe drought stress (LS, (Figure 1a)).

As a consequence of maintaining the soil moisture level at a constant level, irrespective of demand, treatment groups were given very different total water amounts over the course of the experiment. Averaged over plants within treatment groups, the early stress treatments received far greater total water than either control or late treatment groups by the end of the experiment (Figure 1c). This was due to the greater amount of water required to keep the early stress treatment groups at control soil moisture levels (FTSW = 1) during recovery after the end of the early stress period. Additionally, despite markedly lower soil moisture levels (Figure 1b), the LM stress group received slightly more water than the control group by the end of the experiment as well.

**Figure 2:**
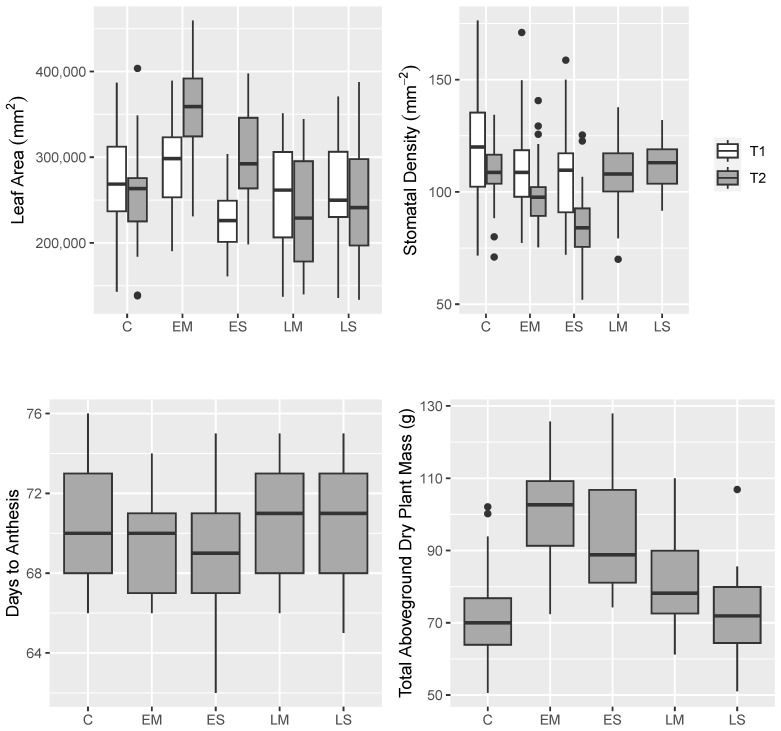
Boxplots of the key phenotypic traits.

Many phenotypic traits varied across drought levels, drought periods, and after recovery (Figures S1 and S2, Tables S1 and S2). Notably, the leaf area was decreased at the end of early stress, yet these same plants exhibited a higher leaf area after recovery at final harvest (Figure 2). Additionally, stomatal density decreased after early stress and this difference persisted and was magnified after recovery (Figure 2). For plants stressed only during the reproductive phase, the leaf area and stomatal density were much more comparable to the control. Surprisingly, the total aboveground biomass was markedly higher in the early stressed plants than in the control, and drought during the reproductive phase only minorly affected the biomass (Table S2, Figure 2). Interestingly, water use patterns for the treatment groups differed with the early stressed plants using more water during the recovery phase (Figure 1c). In terms of WUE (harvest biomass per unit water given), this resulted in comparable WUE for the control, early stressed, and the LM stress groups. LS attained a higher WUE during the reproductive phase (Figure 2).

Time series data were smoothed via general additive models (GAMs), and treatment group GAMs were compared to the control group. Collar diameter was reduced during the early drought phase, but returned to control levels after recovery irrigation. Leaf number, leaf area (hand-measured), and the percentage of leaf senescence was increased in the EM stress group.

For the majority of the phenotypic traits, variance was explained most by genotype (Figure S3). Drought timing is the primary determinant of total water added, as well as traits that are components of biomass (plant weight, capitulum weight).

#### 2.1.2. Trait–Trait Correlations

**Figure 3:**
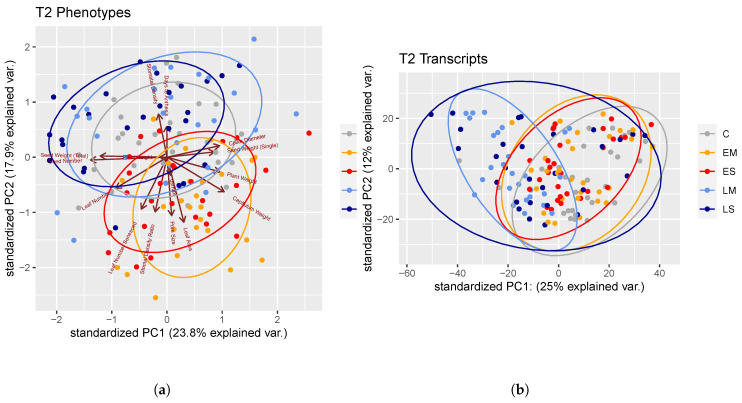
Principal components 1 and 2 from a PCA of phenotypes (**a**) and transcripts (**b**) collected at T2. Ellipses capture the treatment group attribution of the samples.

Comparing the trait differences between treatments in a multivariate (PCA) way showed that trait variation after late drought stress was less diverged from the control than early drought stress (Figure 3a). PC1 primarily separated the early stress treatment groups from the late stress and control groups.

Correlation matrices were altered more due to early drought stress than due to late drought stress (Figure S4). Though all phenotypic correlation matrices were significantly similar according to Mantel tests, the control correlation matrix was more similar to the late matrix than the early matrix (control/late Mantel = 0.61; control/early Mantel = 0.56; early/late Mantel = 0.71). When timing/severity treatment groups were compared, the two least similar correlation matrices were the control and ES (Mantel = 0.49), and the control and EM (Mantel = 0.55).

### 2.2. Transcriptomic Analyses

#### 2.2.1. Differential Gene Expression

**Figure 4:**
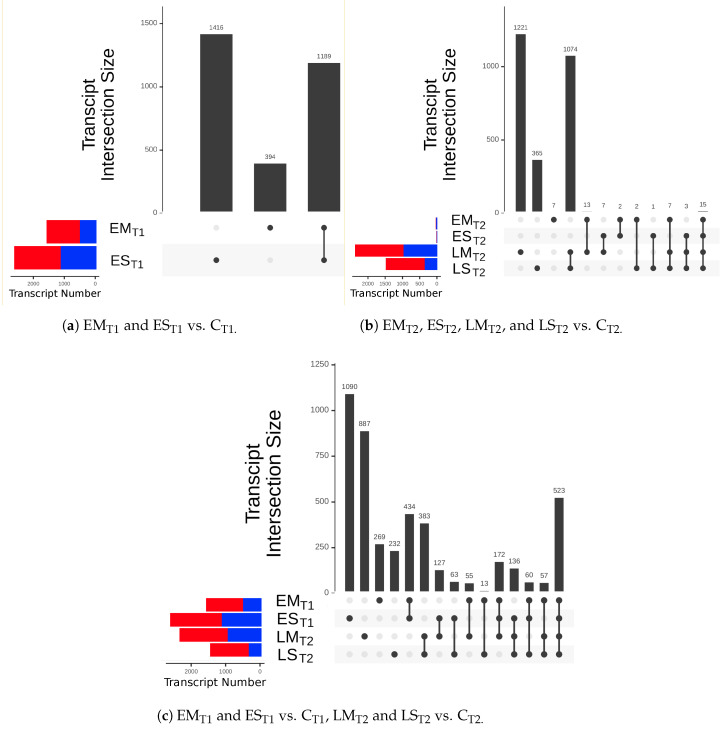
Number of significantly differentially expressed genes (DEGs) in a series of treatment group/time point contrasts. Plants from each stress treatment are contrasted to plants from the control group from the same time point (T1 = vegetative development, T2 = reproductive development). The red and blue portions of the transcripts per contrast bars indicate up- and downregulated transcripts, respectively. (**a**) EM_T1_ and ES_T1_, DEGs between drought stress treatment groups and the control at T1 (**b**) EM_T2_, ES_T2_, LM_T2_, and LS_T2_, DEGs between drought stress treatment groups and the control at T2 (**c**) EM_T1_ ES_T1_, LM_T2_, and LS_T2_, DEGs between drought stress treatment groups and the control at the time of drought stress.

Numerous genes were differentially expressed (DE) in response to drought stress conditions. The magnitude of the differential expression can be seen in UpSet plots (Figure 4) for the various contrasts.

Three contrasts of differential expression were considered. In each of the three contrasts, we used a 1-sample proportions test comparing the proportions of upregulated and downregulated DEGs to determine the directionality of DE during drought stress. In every group, the ratio of upregulated DEGs to downregulated DEGs was significantly greater than the null expectation of 0.5 (*p* < 0.05 × 10^−10^).

The first contrast considered DEGs between the early stress treatments and control at the vegetative phase (EM_T1_ and ES_T1_ vs. C_T1_; Figure 4a). At this time point, there were more DEGs under ES stress than under EM stress. Of the 2991 DEGs in this contrast, 1189 were in both EM and ES, 394 were DE only in EM, and 1408 were DE only in ES. Of the 1189 that were DE in both EM and ES, all but three of these genes differed from the control in the same direction in both stress groups, and most (72%) were more DE in the severe stress group than in the moderate stress group (Table S3). Likewise, there was a greater magnitude (log_2_ fold change) of DE in EM–C contrasts than in ES–C contrasts (Table S4).

The second contrast considered DEGs between stress treatments and the control during the reproductive phase (EM_T2_, ES_T2_, LM_T2_, and LS_T2_ vs. C_T2_; Figure 4b). At T2 (end of the reproductive phase), EM and ES were restored to control levels of soil moisture, whereas LM and LS were drought stressed. At this time, after recovery, EM and ES had very low counts of DEGs (46 and 29, respectively), whereas LM and LS had far greater numbers of DEGs (2340 and 1467 respectively). Of the 1099 that were DE in both LM and LS, all differed from the control in the same direction in both stress groups, and most (57%) were more DE in the moderate stress group than in the severe stress group (an opposite trend from that seen after early stress; Table S3). This inversion of severity effect between early and late drought stress is manifested when looking at the magnitude (log_2_ fold change) of the differential expression in DEGs from LM–C and LS–C contrasts, but not when looking at log_2_ fold change of genes broadly (Table S4).

The third contrast considered DEGs between stress treatments and the control at the time point of the stress (EM_T1_ and ES_T1_ vs. C_T1_, LM_T2_ and LS_T2_ vs. C_T2_, Figure 4c). Although this contrast combines elements of the previous two, it allows us to identify DEGs during both early and late stress. Five hundred and twenty-three genes were DE between all stress groups and concurrent controls, and all but one differed from control expression levels in the same direction between the moderate and severe treatments.

#### 2.2.2. Gene Co-Expression Modules

To cluster gene expression changes into modules, we used CEMiTool. This identified 58 gene modules (and one uncorrelated group). Within these modules, we could identify hub genes that were defined by high intra-module connectivity. The full list of all hub genes is available in the data repository.

**Figure 5:**
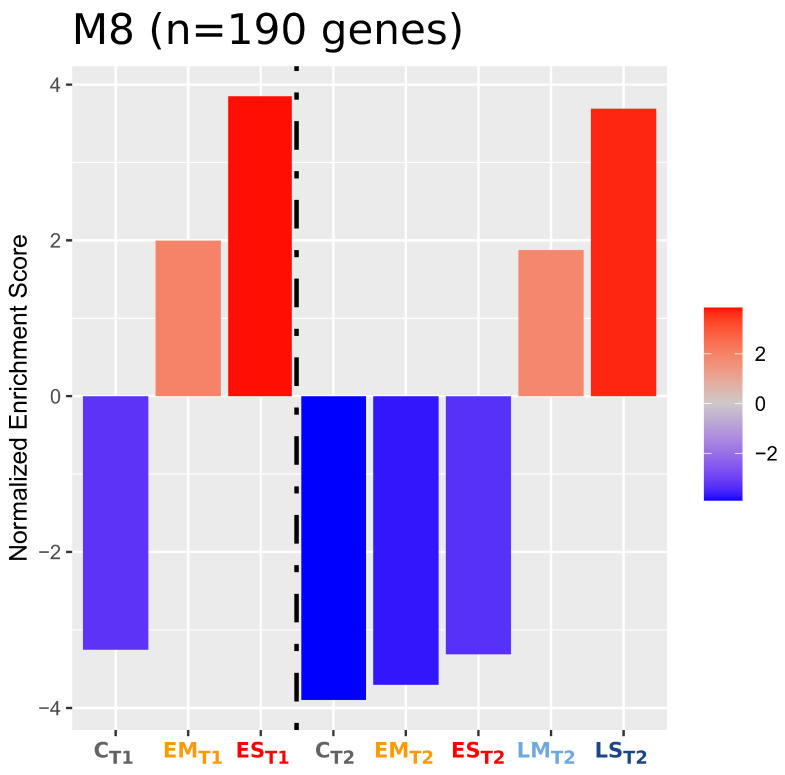
Normalized enrichment scores (NESs) of co-expression Module 8 (M8). M8 is active in abiotic stress responses and proline biosynthesis. The dashed line separates samples at the T1 and T2 time points.

Five modules (M6, M8, M18, M26, M52) were enriched in opposite directions between groups under drought stress (EM_T1_, ES_T1_, LM_T2_, LS_T2_) and groups in either control (C_T1_, C_T2_) or recovery (EM_T2_, ES_T2_) irrigation (Figure S5). Of these modules, the enrichment score of M8 was most strongly correlated with drought conditions. M8 was upregulated during drought stress (more so in severe stress) and downregulated during control and recovery conditions (Figure 5). Conversely, M6, M18, and M52 (and to a lesser extent, M26) were downregulated during stress and upregulated in the control and during recovery.

Nine of the modules had eigengene values that were significantly associated with one or more factors of the experimental design (Figure S6). M8 eigengene value was associated with developmental time point (T1 vs. T2), drought timing (C vs. E vs. L), and the interaction of drought timing and severity (C/E/L vs. M/S). M1 and M3 eigengene values were strongly associated with elements of the experimental design as well (M1, developmental time point; M3, developmental time point and drought timing/severity interaction). No modules were significantly associated with the three-way interaction of developmental time point, drought timing, and drought severity, though two (M1 and M8) were significant at the p<0.05 threshold.

To determine module/phenotype relationships, Pearson correlations between module eigengenes and trait values were hierarchically clustered (Figure S8). M8 was strongly negatively correlated with plant height and days to anthesis, and was moderately positively correlated with seed number, total seed weight, and capitulum weight. Within these module phenotypic correlations, M8 clustered most closely with M1, followed by M25 and M38.

Graphical representations of associations between genes and gene functions were generated for M8 (Figure S9). Most of the enriched gene functions of M8 were closely connected, while a smaller subset of the M8 genes with functions related to proline/glutamine biosynthesis and metabolism were less integrated.

#### 2.2.3. Overrepresentation Analysis (ORA)

Overrepresentation analysis (ORA) was used to relate biological functions related to identified modules. Two modules (M1, M3) had functions related to specific developmental stages. M1 had functions related to pollen recognition and binding (Figure S7a), and M3 had functions related to vegetative growth (Figure S7b).

Modules M6, M8, M18, M26, and M52 were differentially enriched between drought stress and control/recovery groups. M8 genes were overrepresented for numerous stress response functions (heat response, water response, redox homeostasis, protein assembly) and proline synthesis (Figure S7c). M18 was overrepresented for genes with functions related to molybdenum ion binding (Figure S7d), M26 was strongly overrepresented for gene functions related to photosynthesis (Figure S7e), and M52 was overrepresented for genes with functions related to managing reactive oxygen damage (Figure S7f). M6 was not significantly overrepresented for any gene functions.

**Figure 6:**
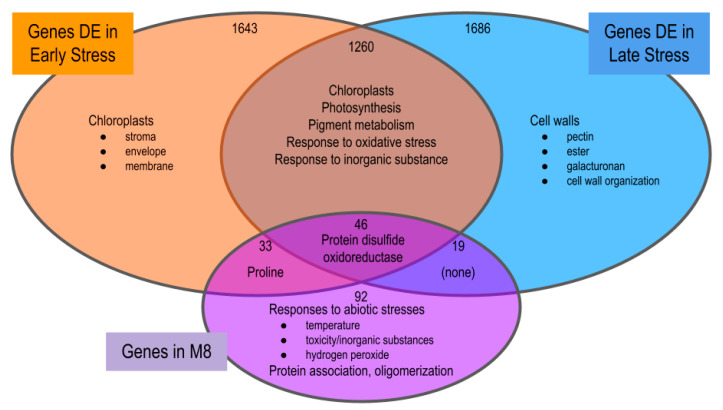
Gene counts and overrepresented gene ontology molecular functions in overlaps of DEGs in E_T1_-C_T1_ (orange), DEGs in L_T2_-C_T2_ (blue), and M8 (purple).

To compare the functions of DEGs involved in early drought stress response, late drought stress response, and members of M8, we partitioned gene sets corresponding to DEGs during early drought stress (E_T1_ vs. C_T1_, n=2982), DEGs during late drought stress (L_T2_ vs. C_T2_, n=3011), and genes in M8 (n=190, Figure 6). Severity was not considered for these contrasts. One thousand three hundred and six genes were DE in both early and late stress, representing 43.8% of early DEGs and 43.4% of late DEGs. Of the 1306 DE genes during early and late drought stress, 46 were also members of M8. These 46 genes were overrepresented for protein disulfide oxidoreductase. The remaining 1260 DEGs in that group were overrepresented for GO functions related to plastid membranes, photosynthetic processes, pigment metabolism, and responses to oxidative stress and inorganic substances. The 33 DEGs during early stress that were members of M8 were overrepresented for proline/glutamine biosynthesis/metabolism. DEGs exclusive to early stress were enriched for chloroplastic functions, DEGs exclusive to late stress were enriched for functions related to cell wall metabolism and maintenance, and members of M8 that were not DE in either stress treatment were overrepresented for functions related to the response to various abiotic stresses (temperature, toxicity, water deficit, hydrogen peroxide) and protein oligomerization.

## 3. Discussion

### 3.1. Similar Transcriptomic Responses to Early and Late Drought Stress Result in Divergent Phenotypic Outcomes

Here we show that drought stress responses in sunflower vary greatly depending on the timing of drought onset and, to a lesser extent, drought severity. We used an automated high throughput phenotyping platform (Heliaphen [31]) to subject six cultivated sunflower lines to two drought severities at two developmental time points (allowing for recovery of vegetative phase stressed plants). Though similarities in plant responses exist between all four drought conditions, plant phenotypic responses to drought during earlier vegetative growth (T1) were notably different from responses to drought during later reproductive growth (T2). While others [32] have pointed out that similar phenotypic abiotic stress responses may be reached via divergent transcriptomic routes, our water limitation experiment has revealed an inverse pattern: Sunflowers responding to drought stress early or late in development utilize similar transcriptomic responses, yet these result in divergent phenotypic outcomes.

### 3.2. Divergent Phenotypic Outcomes

#### 3.2.1. Water Use and Biomass

Contrary to expectations based on other studies with similar methodology [27], plants subjected to EM drought stress accumulated more biomass than plants subjected to late drought stress or control plants when allowed to recover after drought. This difference was more pronounced in the EM drought stress than in the ES drought stress. In addition to the greater biomass, the EM group also had greater leaf area and leaf number than the control group.

This differential growth pattern was likely linked to the fact that EM and ES plants received far greater total amounts of water (roughly a liter and a half more over the control group, equivalent to roughly 233.2 mm of extra irrigation or precipitation, or the volume of soil in each pot) over the course of the experiment. Maintaining early stressed sunflowers at control soil moisture levels during recovery irrigation thus required far greater volumes of water. Though root phenotype data were not collected in this experiment, a possible explanation is that the early groups developed larger root systems during stress, which resulted in the capacity for rapid water uptake during recovery irrigation, more rapid pot dry-down, and greater water provisions to maintain predetermined levels of soil water moisture. This greater water flow across the root system could then have resulted in greater nutrient uptake and enhanced growth.

Root development has been identified as a key factor in drought stress tolerance for sunflowers [30,33], as well as *Arabidopsis* [34], maize [35], common bean [36], soybean [37], wheat [38], cotton [39], and rice [40]. Plants frequently exhibit plasticity in resource allocation in such ways as to increase the capture of scarce resources [41]. Plants that allocate proportionally greater resources to the development of organs that maximize acquisition of limiting environmental resources greatly increase biomass [42]. Sunflower is known to increase root mass fraction to increase water use efficiency when water is scarce [33,43]. While shoot length is frequently inhibited by drought conditions, root growth (particularly root apex expansion [44]) is less inhibited, and sometimes promoted, by low soil water potential [35,45,46,47] unless drought severity is too great [30]. While plants in late stress (especially LS) demonstrated elevated WUE, those in early stress did not. Early stressed plants consumed nearly 150% of the water of the control group and displayed commensurately higher plant weight. LS plants, moreover, were provided significantly less water than the control plants, but reached the same dry mass as in the control. Though we must speculate about the subsoil development of the sunflower plants in our study, higher root mass ratio would permit greater rates of water uptake upon recovery irrigation, which would explain (at least in part) the observed overcompensation, i.e., greater biomass in response to early drought stress.

While this system of irrigation (and, therefore, plant growth response) may seem unnatural at first, it may mimic to some degree the experience of plants (such as sunflower [48], rice [40], and wheat [49]), that develop more extensive root systems under drought conditions so as to reach deeper water reserves. Therefore, the degree to which this study demonstrates applicable plant stress response dynamics depends on the plant system in question and the agronomic environment to which the comparison is applied.

#### 3.2.2. Leaf Traits

An alternative/parallel explanation for elevated water demand in early stressed plants could be that leaf morphology was modified, resulting in lower WUE and greater water loss. However, leaf phenotypic data do not support this explanation. Early plants exhibited reduced $g_{smax}$ due to lower stomatal density and smaller pore size, suggesting that the leaves were conditioned for higher WUE, not lower. Higher WUE is generally correlated with decreased $g_{smax}$ [50,51], reduced stomatal size, and increased stomatal density [52,53,54], though this pattern is variable between plant lineages and differences in drought stress timing and severity [55,56]. For example, in the grass *Leymus chinensis*, moderate drought stress induces increased stomatal density, whereas severe drought stress induces lower stomatal density [54].

While droughted leaves appeared to have a morphology consistent with high WUE, it remains possible that early stressed plants had a higher water demand due to increased water loss arising from an increase in aboveground transpirational surface area. Studies have found that sunflowers exhibit reduced leaf area as a plastic response to drought stress [22,24,27], though opposite trends are found by Takami et al. [25] and Rawson and Turner [57]. Contrary to those expectations, the EM group exhibited a higher leaf count and leaf area than the control, and only the ES group had lower leaf counts and area in response to early stress.

Upon recovery, early stressed plants had a greater leaf area and number than the control plants. This increase in leaf area and number was accompanied by an increased percentage of leaves senesced during recovery irrigation. Leaf number is negatively correlated with leaf number senesced within treatment groups, but neither of these traits are strongly (positively or negatively) correlated with the total leaf area, possibly because the leaf area was more influenced by leaf size or leaf wilting not resulting in leaf senescence. It is possible that greater leaf expansion is due to greater cell expansion resulting from drought stress during the early stages of leaf development [58]. In total, these data present the picture of EM plants achieving a higher leaf surface area than the control during recovery irrigation despite a higher average leaf loss.

#### 3.2.3. Photosynthesis

Transcriptomic data strongly suggest a role of photosynthesis regulation in response to drought in sunflower. DEGs in both early and late stress were strongly enriched for functions related to photosynthesis directly as well as to chloroplast and thylakoid membranes. Plant growth rate is dependent on photosynthetic rate, and drought stress impairs photosynthesis in multiple ways, chiefly by limiting CO_2_ import into the leaf, but also by damaging photosynthetic molecules and reducing photosynthetic leaf area via leaf senescence and reduced leaf area [55,59]. Previous sunflower drought stress studies have likewise found that drought stress hindered photosynthesis [60] and identified a strong transcriptomic response in photosynthesis related genes [24].

#### 3.2.4. Trait–Trait Correlations

A PCA analysis showed the separation of individuals in trait space due to both drought treatment groups (mostly across PC1, 22.2% of explained variation) and genotype groups (across both PC1 and PC2, 14.7% of explained variation). Moreover, our data showed that early stressed plants were more phenotypically diverged from the control than the late stressed plants. The PCA placed early plants shifting away from the control and late plants across PCs 1 and 2. Pairwise trait–trait correlations were mostly preserved regardless of drought treatment conditions (trait–trait correlation coefficient matrices were significantly similar according to Mantel tests). However, analyses showed that early stress caused greater divergence from control-like trait–trait correlations than did late stress. Taken together, these results illustrate that early stress caused a greater phenotypic change than did late stress.

### 3.3. Similar Transcriptomic Responses

#### 3.3.1. Differentially Expressed Genes

Different genes are involved in the early and late drought stress response, and in moderate (40% transpirable soil water) vs. severe (20% transpirable soil water) stress (Figure 4). Despite general differences in DEGs between treatments and time points, in many respects, drought severity acts as a “volume knob” for transcriptomic response. At the vegetative time point (T1), differential expression was in the same direction for both moderate and severe stress, but the magnitude of DE was greater with severe stress. Conversely, at the reproductive time point (T2), expression was still in the same direction in moderate and severe stress, but moderate stress elicited a greater expression response. A possible explanation for this inversion could be that, during the later drought period, severely stressed plants are stressed to such an extent that they cannot mount a sufficient response. However, our data do not support this explanation, as the early and late stressed groups had similar numbers of DE genes, indicating that gene expression was just as pliable at T2 as at T1.

For significant DEGs between the drought stressed and control groups, more DEGs were upregulated than downregulated. This replicates the findings of Liang et al. [61], who also found more upregulation than downregulation in sunflower drought response.

In general, patterns of strong differential expression during early stress returned to control-like levels after recovery irrigation. Given the sampling at the end of the reproductive phase, we have to note the possibility that we missed an earlier window during stress recovery in which recovery genes were DE for a time before returning to control-like levels of expression. Differential expression 36 h after recovery irrigation has been documented in sunflower [17]. Our data indicate that these experimental drought conditions did not initiate long-term transcriptomic alterations, and that any patterns of differential expression that may have been involved in post-rehydration recovery returned to control-like expression patterns by harvest at T2. We also note that, even if DEGs in the early drought stressed plants reset to control-like expression levels after recovery, other types of responses (protein, metabolomic, morphological) are less transient and may persist longer, which may account for the persisting phenotypic divergence.

We note that this study’s methodology may result in a disconnection between soil moisture and other environmental factors, such as evaporative demand, temperature, and UV-B radiation, frequently correlate with soil moisture. In our study, all plants experienced similar ambient temperature and humidity, despite differences in soil water content. This means that there were likely plants with dry air and wet soil, and perhaps vice versa. This is a departure from natural conditions, and may influence trait development or behavior. For example, stomata behavior responds to humidity in such a way as to reduce water loss [62]. Some drought stress responses are contingent upon or modified by concurrent abiotic stress signals [63,64]. Likewise, the experimental design of this project involved water limitation stress without elevated heat stress, as would be expected given natural drought events. Several sunflower studies have shown that stress combinations have non-additive effects on plant response [32]. While stomatal closure reduces water transpiration, it also reduces heat dissipation [65]. Therefore, there was likely a diminished fitness cost of closed stomata. These concerns are, however, somewhat ameliorated by strong correlations between phenotypes in field and Heliaphen environments documented by Gosseau et al. [66].

#### 3.3.2. Co-Expression Networks

Though we identified 58 gene modules, only two (M8, M26) showed a role in drought stress response (Figure S5). Module M26 contained 102 genes and displayed a weaker signal of drought response. M26 was enriched during drought stress groups (excepting LS) and under-enriched during control/recovery conditions. This module was strongly overrepresented for many of the same photosynthetic and plastid gene functions as identified in the early and late DEGs, yet it has very little overlap with genes identified via DE analysis.

The module most strongly associated with drought stress response was module M8 (containing 190 genes). M8 was enriched during drought stress, and more so under severe drought stress. Interestingly, this module was overrepresented for numerous gene functions related to a wide range of abiotic stress responses, indicating its potential association with responses to multiple types of stress.

Within module M8, we identified three hub genes. The top hub gene, gene: Ha412HOChr02g is a putative ricin B-like lectin (R40G3, LOC118488102). Lectins are carbohydrate-binding proteins that perform myriad functions [67]. Some lectins, such as ricin B-like lectins, are implicated in drought stress response [68,69]. Moreover, R40G3 has been identified as highly DE in drought stress experiments in barley [70] and wheat [71]. This gene was DE in early stress but not after late stress. The second hub gene, mRNA: Ha412HOChr07g0304341, a putative dehydrin (Xero 1, LOC110867930), is also DE in early stress but not during late stress. Dehydrins have previously been identified as actors in sunflower drought stress response [17,23,61]. The third hub gene, mRNA: Ha412HOChr03g0139171, is DE in neither early stress nor during late stress, and is a putative small chloroplastic heat shock protein (HSP20, LOC110930642). Heat shock proteins (HSPs) are molecular chaperones that guide the protein folding during translation of many kinds of environmental stress, including (but not limited to) heat [72]. The functions of the three hub genes of M8 further illustrate the clear role of M8 in drought stress response.

In addition to the hub genes’ stress functions above, M8 was overrepresented for genes active in the proline biosynthetic pathway. Proline accumulation is a common response to various abiotic stresses for many plants [73,74,75,76], including sunflowers [24,58,77]. This adds further support to the role of M8 in the drought response.

Overrepresentation analysis revealed that the primary function of genes at the intersection of early DEGs, late DEGs, and M8 genes, was the maintenance of photosynthetic systems. This three-way intersection between early, late, and M8 had only 46 genes and was overrepresented for a single term, protein disulfide oxidoreductase activity. Oxidoreductases are enzymes that generate disulfide bonds, a critical function in the folding and assembly of proteins during protein synthesis [78]. Disulfide bond formation is particularly important during photodamage, when photosystem II requires rapid reassembly of thylakoid membranes [79,80].

Concerning drought timing, the DEGs shared by the early and late stress were overrepresented for functions related to plastids and photosynthesis, as well as other stress response mechanisms. DEGs after late stress were additionally overrepresented for genes that functioned in the biosynthesis of cell wall components. A possible mechanism behind this may be that late plants experienced drought stress in the form of extra turgor pressure and responded by strengthening leaf cell walls. These cell wall modifications can serve as drought stress adaptations in some plants [81], possibly by preventing leaf damage during desiccation [82].

### 3.4. Compensatory and Overcompensatory Growth

Compensatory effects are post-stress growth and development that compensate for growth and development lost or delayed due to stress. When post-stress growth results in greater trait values than would be achieved without stress conditions, this is known as overcompensation. The degree and prevalence of compensation and overcompensation can vary by genotype/cultivar [39], by stress type, severity, duration, and developmental timing [83], and by other concurrent environmental conditions [84]. Our results here point toward overcompensation in response to early drought stress for traits related to aboveground biomass and leaf area.

Two studies in sunflower [25,57] identified cases in which particular sunflowers exhibited overcompensation in the leaf area following drought stress, though the degree of compensatory effects varied between studies and between cultivars. Overcompensation of drought stress and subsequent recovery irrigation on plant biomass and tissue development have also been reported in [39], soybeans [76,83,85], potatoes [86], and Chinese rye grass [87]. The results of our work point toward overcompensatory effects of early drought stress on traits related to aboveground biomass and leaf area. While early plants achieved greater biomass and area vs. control, this was accompanied (effected) by greater water acquisition, reflecting greater water demand, and ultimately similar WUE. Despite their taxonomic distance from *Helianthus*, some vertebrates have demonstrated a similar pattern with overcompensatory hyperphagia. For example, Dulloo [88] showed that humans (*Homo sapiens*), when malnourished, will undergo hyperphagic overcompensation to greatly increase their body weight upon the return of liberal food supplies (post-starvation). Hayward et al. [89] showed a similar pattern in hybrid sunfish (*Lepomis*), and went a step further to show that the gross growth efficiency was unchanged under hyperphagia, meaning that the biomass differences were simply due to eating more. In the same way, sunflowers subjected to early drought stress altered their morphology/physiology/behavior so as to acquire more water upon recovery irrigation, leading to commensurately greater plant size.

We further clarify that the overcompensatory growth observed in this study was not found to result in higher yield. Though yield data were collected, our collection methods did not allow for accurate comparisons between treatment groups. We observed that late stressed plants unexpectedly exhibited higher yield, which we speculate was due to more rapid seed filling. Plants may allocate greater proportions of their resources to reproduction, so as to mitigate the loss of reproductive output [41]. Though drought stress generally inhibits seed filling by inducing a shorter seed filling period [43,90,91,92,93,94], drought stress may result in more rapid seed development in the short term [94] (though not all studies bear this out [92]).

We must also separate the ideas of overcompensation of a suite of traits (plant size and biomass) and overcompensation in fitness (plant survival and reproduction). Even beyond the context of overcompensatory growth, it is difficult to establish fundamental connections between traits and fitness. If the overcompensatory growth demonstrated by the early group is reflective of higher fitness than that displayed by plants in the control group, it leads one to question the nature of “stress” and “control” groups in this context: If drought treatments lead to higher fitness, is it truly stress? Past studies have used water deficits of this severity and lower to successfully stress sunflowers [30], so we conclude that plants were likewise stressed in this study.

Nevertheless, we believe that higher plant biomass is suggestive of higher yield, as has been shown in various other crop systems such as maize [95] and soybean [96]. Furthermore, Rawson and Turner [97] found that leaf area and yield were strongly correlated in sunflowers. Some authors promote the potential utility of employing compensatory effects in agronomic practice [76,83]. In particular, Rawson and Turner [97] suggested that irrigation might be safely withheld from sunflowers for a “considerable portion” of their vegetative growth when subsurface soil moisture is sufficient, thanks to sunflower’s capacity to compensate during recovery.

These results illustrate that sunflowers can respond to even severe water deficits via developmental modifications that situate the plant for greater recovery and high growth rates, possibly resulting in higher long-term fitness and yield. We recommend that future studies be designed to further investigate the possibility/utility of overcompensation increasing performance. If overcompensatory growth indeed results in higher performance (which is likely true for crops in which biomass is strongly associated with yield), then subjecting crops to similar drought/recovery irrigation schemes may prove beneficial when water resources are abundant and where the timing and quantity of water application can be controlled.

### 3.5. WUE, EUW, and Drought Stress Response Strategies

Overcompensatory growth was not associated with greater WUE, but rather with maximizing soil water acquisition. The capacity to capture maximal amounts of water and use it for transpiration was termed by Blum [98] as “effective use of water” (EUW). WUE, here defined as grams in dry biomass per unit of water, is more affected by changes to water use than to changes in biomass [99] and, therefore, higher WUE is frequently achieved by limiting water use by slowing transpiration and productivity [49]. Conversely, a plant with high EUW will develop tissues to maximize water acquisition and maintain high stomatal conductance and transpiration through drought conditions, while limiting non-productive water loss through non-stomatal transpiration and soil water evaporation. Sunflowers have been labeled “drought avoiders” [29] for their proclivity to continue carbon assimilation through drought stress by allocating resources to gather water resources via deep root systems [21,29]. Sunflowers have also been accused of “profligate” [100] water use due to soil water depletion and high and insensitive stomatal conductance, though closer examination reveals that sunflowers have WUE comparable to other C3 plants, because they can modify their water expenditure by controlling their leaf area [101], a capacity shown by the early stressed plants in this experiment. For the purposes of plant breeding, EUW may be more important than WUE–but see the maize line Michoacan-21 and the *latente* trait [102,103] for an example of successful drought stress resistance via delayed growth.

Plants in the late stress group (particularly LS) demonstrated increased WUE. The LS plants were provided significantly less overall water without suffering significant decreases in biomass (plant weight, capitulum weight) relative to control. The late groups also exhibited higher yield, though, as discussed above, confounding variables may account for this apparent difference.

## 4. Materials and Methods

### 4.1. Experimental Design

#### 4.1.1. Common Garden

Six sunflower maintainer (B) lines bred for oil-rich seeds from the SAM population [104] (HA124, HA370, HA412HO, HA850, HAR4, and SF193, also known as XRQ) were grown at the Heliaphen outdoor high-throughput phenotyping platform at INRAE Toulouse (France) in 2018. More details of the Heliaphen phenotyping platform are outlined by Blanchet et al. [31]. Seeds were planted on 17 April 2018 in 10 L pots of Terreau Proveen PAM 2 substrate. Plants were fertilized with 300 mL of Peter’s Professional 17-07-27 and 200 mL of Hortrilon per pot on 5 May, 25 May, and 13 June, and were provided Ortiva Top fungicide on 28 May and 15 June. Pots were maintained at specified soil moisture levels by measuring the pot weights daily and refilling them with water to return the pot weights to target levels. Soil around the plants was covered with a silicone cover to limit soil evaporation and was protected from rain using a cone-shaped polystyrene skirt. Drought treatment regimes were imposed by permitting pot weights to drop to specified levels for set periods of time.

Plants were grown under one of five irrigation treatment groups (Figure 1a). Control plants were provided daily water to maintain a pot weight equal to the recorded weight of the pot left to drain for two hours after full water saturation [31], defining a fraction of transpirable water (FTSW) of 1. The early moderate (EM) and early severe (ES) drought stress groups were subjected to drought stress during the vegetative growth phase from 7 May until target stress levels (EM = 0.4 FTSW; ES = 0.2 FTSW) were reached (Figure 1b). Early drought stress target soil moisture levels were reached between 1 June and 11 June. Following these early drought stress treatments, these plants were returned to and maintained at control levels of soil moisture (FTSW = 1) until the end of the experiment. The late moderate (LM) and late severe (LS) drought stress groups were subjected to drought stress during the reproductive growth phase starting on 12 June (plants initiated flowering between 18 June and 2 July). Late drought stress was maintained at target drought levels (LM = 0.4 FTSW; LS = 0.2 FTSW) until 3 August, after which all plants were denied irrigation and left to dry until harvest on 31 August. Thus, treatment groups for this study were control (C), early moderate (EM), early severe (ES), late moderate (LM), and late severe (LS). Six replicates of each combination of treatment group (*n* = 5) and genotype (*n* = 6) were grown, resulting in 180 plants, of which 174 survived.

The volume of water provided daily to each plant was measured automatically, permitting us to track the time series of water provision to each plant in each treatment group (Figure 1c). One potential source of error introduced by this irrigation regime is that not all water provided to the plant was transpired. Some small but unmeasured portion of the water inevitably evaporated from the soil surface despite the use of silicone pot covers. More notably, error was introduced by water being taken up and stored in the plant. Water stored in the plant increases the pot weight, reducing the amount of water the automated system will provide in subsequent rewatering events, resulting in a soil moisture lower than targeted levels. The scale of this effect is, however, likely to be minimal due to the large difference in weight between the pot/soil and plant biomass.

#### 4.1.2. Data Collection

Several phenotypic traits (plant height, leaf number and area, collar diameter) were measured multiple times over the course of the experiment, producing the time-series data. Because the phenotyping platform (RapidoScan RS-C-025-1600-MOD) records a maximal plant height of 1.5 m, plant height data were truncated to those collected before 28 June, the date when some plants began to reach this height. Total leaf area was calculated for each plant by finding individual leaf areas for every other leaf following the leaf area equation from [105] and doubling the sum of those values.

Other traits were collected at the end of the experiment after harvest and after 48 h dry-down at 80 °C for yield components (seed number and weight, plant weight). Aboveground plant weight was divided into total seed weight, capitulum weight, and vegetative (leaf and stem) plant weight. Total water added was defined as the total water provided to a single plant over the course of the experiment. We defined water use efficiency (WUE) as the total dry plant weight (vegetative plant weight plus capitulum weight plus total seed weight) per total water added per plant.

At the end of each stress treatment (12 June and 3 August, respectively), dental putty imprints of both sides of leaves one third of the way down the plant were taken for stomatal data collection following the methods of Earley et al. [106]. Clear nail polish was applied to the dental putty imprints and removed with clear tape. Images were taken from three or four 100× magnified areas per leaf print using a light microscope. Stomata were counted manually for each image and averaged per leaf side. Ten individual stomata per leaf side were imaged at 400× and measured for pore length and guard cell width (as a proxy for pore depth). Using these stomatal measurements (density, pore size, pore depth), we calculated the maximum anatomical stomatal conductance ($g_{smax}$ hereafter) following the diffusion equation in Dow et al. [107] (originally derived from Parlange and Waggoner [108] and Franks and Farquhar [109]). It should be noted that, given the duration and implementation of our drought treatment, the first sampled leaves began development before the early drought stress and finished during the early drought stress period, though the second sampled leaves fully developed during the drought stress period.

### 4.2. RNA-Seq Pre-Processing and Transcript Quantification

The leaves opposite those selected for stomatal analyses were sampled for RNA extraction at two time points (T1 and T2), corresponding to the end of each stress treatment (12 June and 3 August, respectively). RNA was not sampled from LM and LS at T1, as their treatment was identical to C at that time point. RNA libraries were prepared and sequenced at the University of Georgia Genomics and Bioinformatics Core using the Kapa Biosystems library preparation chemistry and sequenced on an Illumina NextSeq with 75 bp single-end reads. Replicates for each genotype/treatment combination were spread across three multiplexed sequencing pools.

Sequences were processed using a custom-built pipeline available at https://github.com/EDitt/Sunflower_RNAseq, accessed on 23 September 2020. Transcripts were assessed for quality with FastQC/MultiQC [110,111] and trimmed of adapter sequence with Trimmomatic [112]. Reads were mapped to the Ha412HOv2 reference genome with STAR [113] following a two-pass mapping strategy that identifies novel splice junctions with greater sensitivity, and transcript quantification was conducted with RSEM [114].

RNA-Seq data from samples with a low mapped count coverage (mean transcript count < 3) were discarded from the RNA-Seq analyses. In total, from a maximal possible set of 288 samples, 231 samples met the inclusion criteria.

### 4.3. Phenotypic Analyses

#### 4.3.1. Phenotypic Means and Variance

The following mixed-effect linear model (R function lmer, R package lme4 [115]) was used for the purpose of detecting differences in the phenotypic means between treatment groups:

(1) TRAIT∼TIMING+TIMING:SEVERITY+(1|GENOTYPE)

In this model, trait represents the values of a given trait, timing indicates early or late stress, severity indicates moderate or severe stress, and genotype is the random effect of the sunflower line. Severity is conceived of as a nested effect within timing, rather than as a main effect.

The following linear model (R function lm) was used for the purpose of attributing the phenotypic variance to elements of experimental design:

(2) $$\begin{array}{c} TRAIT∼TIMING+TIMING:SEVERITY+GENOTYPE+\\ GENOTYPE:TIMING+GENOTYPE:TIMING:SEVERITY. \end{array}$$

In this model, timing indicates early or late stress, severity indicates moderate or severe stress, and genotype indicates sunflower line. Whereas the sunflower line was treated as a random effect in the previous model, we include it here as a fixed effect so as to determine the amount of variance attributable to genotypic differences. One-way ANOVA (R function anova) was used to find the sum of squares for each term in the model.

For time series trait data, general additive models (GAMs) were used to determine the difference in patterns between treatment groups. These GAMs were designed with the following model:

(3) TRAIT∼CONDITION+s(DATE)

In this model, condition is the drought treatment group and s(DATE) is a smoothed function of date. Confidence intervals (95%) around GAMs permit identification of time periods wherein time-series trait values differ between treatment groups.

#### 4.3.2. Trait–Trait Correlations

A principal components analysis (PCA; R function prcomp) was run on phenotypic data collected at harvest. Traits were scaled and centered, and ellipses were included to denote either treatment group or genotype group membership. Phenotypic correlations were calculated with Pearson’s correlation coefficient on a treatment level-specific basis. Traits were clustered according to similarity in correlations with other traits using Ward’s minimum variance (R function hclust, method “ward.D2”), resulting in hierarchical dendrograms. Similarity of treatment group-specific correlation matrices were ascertained with the Mantel test (R function mantel, R package vegan [116]).

### 4.4. Transcriptomic Analyses

#### 4.4.1. Differential Gene Expression

Differentially expressed genes (DEGs) were identified with DESeq2 (R package DESeq2 [117]) using the model:

(4) $$\begin{array}{c} EXPRESSION∼CONDITION*GENOTYPE \end{array}$$

In this model, the condition is drought treatment and the genotype indicates the sunflower line. Significance was determined with the Wald significance test, after the Benjamini–Hochberg correction for multiple comparisons.

To investigate the impact of drought stress on the overall expression, we compared the number of DEGs that were upregulated (positive log_2_ fold change) and downregulated (negative log_2_ fold change) in each drought stress treatment group. A 1-sample proportions test (R function prop.test) was used to determine if the proportion of genes with positive log_2_ fold change values was significantly different from the null hypothesis of equal proportion of upregulated and downregulated genes (proportion = 0.5).

To investigate the relationship between the drought stress severity and degree of transcriptomic response, we compared the number of genes in which (the absolute value of) log_2_ fold change (relative to the concurrent control group) was higher in the severe stress than the moderate stress to the number of genes in which the opposite was true (greater log_2_ fold change in moderate stress than in severe stress). This test was carried out separately for early and late stress groups, as well as for all genes and exclusively DEGs. Divergent genes, i.e., genes that had log_2_ fold change values of opposite signs in moderate and severe treatment groups, were excluded from this analysis. A 1-sample proportions test (R function prop.test) was used to determine if the proportion of genes that had greater DE under severe stress was significantly different than the null hypothesis of a proportion = 0.5.

To further investigate the relationship between drought stress severity and the degree of transcriptomic response, we compared the per-gene difference in (absolute value) log_2_ fold change (relative to the concurrent control group) between moderate and severe drought stress. This test was carried out separately for early and late stress groups, as well as for all genes and exclusively DEGs. Divergent genes, i.e., genes that had log_2_ fold change values of opposite signs in moderate and severe treatment groups, were excluded from this analysis. A paired-sample two-tailed Student’s *t*-test (R function t.test) was used to determine if the difference in log_2_ fold change between moderate and severe stress groups was significantly different than the null hypothesis of 0.

#### 4.4.2. Gene Co-Expression

We used CEMiTool [118] to assemble the co-expression network modules. CEMiTool finds the similarity between pairs of genes via an algorithmically selected soft thresholding power β. Then, the gene groups are separated into modules with dynamic tree cut [119].

To determine how the modules were regulated between treatment/timing groups (C_T1_, EM_T1_, ES_T1_, C_T2_, EM_T2_, ES_T2_, LM_T2_, LS_T2_), we performed a gene set enrichment analysis (GSEA) with CEMiTool (R package fsea [120]). For each treatment group, the gene expression for each gene was averaged across individuals and then ranked using the z-score normalized average expression. This method calculates a running enrichment score along the ranked gene list, which is normalized to produce normalized enrichment scores (NESs). Significance of the NES is determined via permutation test (10,000 permutations) and adjusted for multiple comparisons via the Benjamini–Hochberg correction [121].

CEMiTool identifies hub genes within modules. Every gene in a module has an associated connectivity value, which is the sum of adjacency values with all other genes in the module [122]. Though CEMiTool by default defines the top five genes with the highest connectivity in each module as hub genes, we plotted the gene connectivity and found the elbow in the plot (R function elbow_point, R package akmedoids [123]). In practice, this criterion for hub gene selection is on average slightly less conservative than the other two methods (elbow method mean hub gene percent = 7.1%; top five genes as hub genes mean hub percent = 5.5%; greater than two standard deviations mean hub percent = 3.2%; data not shown).

A module eigengene is the first principal component of the module’s expression matrix. To determine if the gene modules were significantly associated with factors of the experimental design (drought timing, drought severity, developmental time point), we used an ANOVA framework to find if eigengene values varied significantly across those factors or their interactions. We used the following model:

(5) $$\begin{array}{c} EIGENGENE∼TIME\_POINT+TIMING+TIMING:SEVERITY+\\ TIME\_POINT:TIMING:SEVERITY+(1|GENOTYPE). \end{array}$$

We used a similar model to determine if eigengenes were associated with phenotypic traits:

(6) $$\begin{array}{c} EIGENGENE∼TRAIT_{1}+TRAIT_{2}+\ldots +TRAIT_{N}+\left(1|GENOTYPE\right). \end{array}$$

In both models, time point indicated first or second RNA sampling (T1 or T2, respectively), and genotype was included as a random effect.

#### 4.4.3. Overrepresentation Analysis (ORA)

Gene ontology (GO) terms were obtained from annotations of version 2.0 of the Ha412 sunflower genome (Ha412HOv2.0-20181130.gff3). These terms were ordered with go2gmt online tool (http://bioinformatics.sdstate.edu/go2gmt/, accessed on 9 September 2020) to produce a .gmt gene set file. An overrepresentation analysis (ORA, function enricher, R package clusterProfiler [124]) was run for select sets of DEGs, all gene modules, and select gene set overlaps of interest. Graphical representations of associations between genes and gene functions were then generated with cnetplot [125]).

## 5. Conclusions

Drought stresses of variable timing and severity can result in similar transcriptomic responses but highly divergent phenotypic outcomes. Genes differentially expressed in early and late drought stress are overrepresented for functions relating to photosynthesis and plastid maintenance, and a core module of genes is strongly associated with drought stresses. Yet, plants subjected to early stress were phenotypically divergent from those subjected to late stress. We identify a capacity for early-droughted sunflowers to acquire and transpire a much greater amount of water upon recovery irrigation and to have an elevated growth rate commensurate with that increase in transpiration. We speculate that this capacity for rapid water acquisition is enabled by changes in plant architecture (e.g., greater root development during early drought stress, greater leaf area during recovery irrigation). While it is uncertain to what degree or extent this plant response could be leveraged in agronomic settings, overcompensatory growth rates in response to early severe drought stress provide an interesting avenue for research. More broadly, our results underscore the importance of careful consideration when choosing methods to subject plants to water stress in drought studies.

## Data Availability

Data for this study are available at Dryad Digital Repository, https://doi.org/10.5061/dryad.v6wwpzh20, and with BioProject accession number PRJNA976033 in the NCBI BioProject database, https://www.ncbi.nlm.nih.gov/bioproject/.

## Supplementary Materials

The supporting information can be downloaded at: https://www.mdpi.com/article/10.3390/ijms24119351/s1.

## Abbreviations

C	Control
DE	Differentially expressed/Differential expression
DEG	Differentially expressed gene
E	Early
EM	Early moderate
ES	Early severe
EUW	Effective use of water
FTSW	Fraction of transpirable soil water
GAM	General additive model
GO	Gene ontology
$g_{smax}$	Maximum stomatal conductance
L	Late
LM	Late moderate
LS	Late severe
NES	Normalized enrichment score
ORA	Overrepresentation analysis
PCA	Principal component analysis
T1	Timepoint 1
T2	Timepoint 2
WUE	Water use efficiency
