# Supplementary material for: Similar Transcriptomic Responses to Early and Late Drought Stresses Produce Divergent Phenotypes in Sunflower (Helianthus annuus L.)
Source: Int J Mol Sci. 2023 May 27;24(11):9351. doi: 10.3390/ijms24119351 (PMC10253505; doi:10.3390/ijms24119351)
Supplement: Supplementary file 1 [file ijms-24-09351-s001.zip › ijms-2416501-supplementary.pdf]

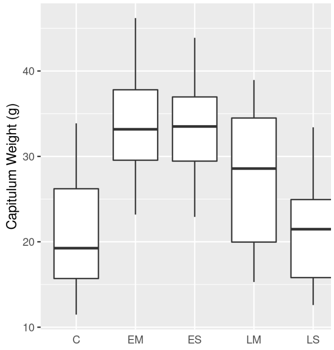

(**a**)

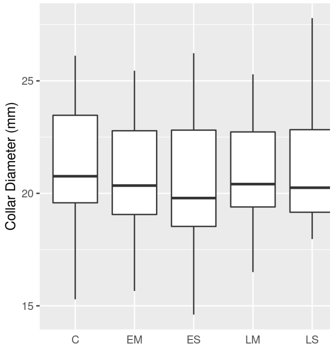

(**b**)

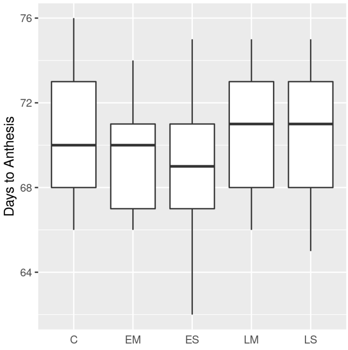

(**c**)

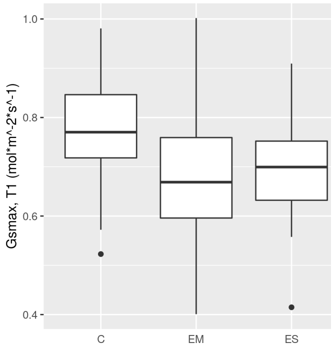

(**d**)

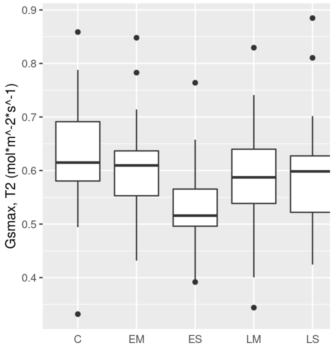

(**e**)

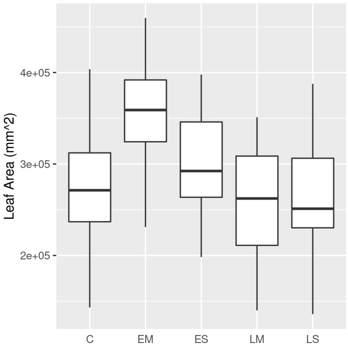

(**f**)

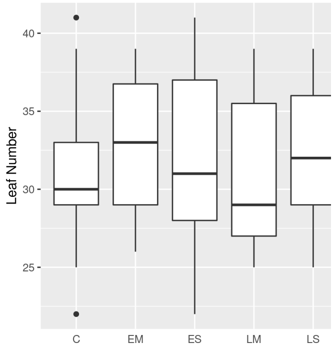

(**g**)

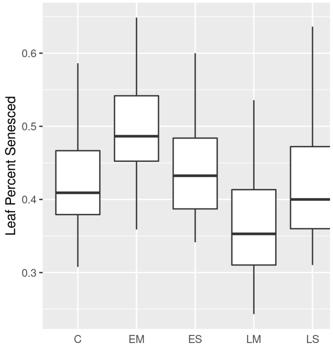

(**h**)

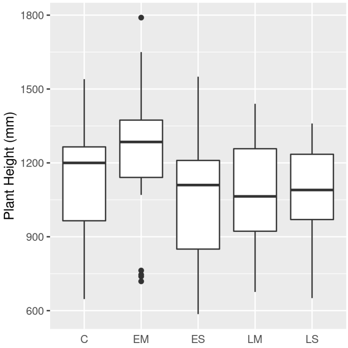

(**i**)

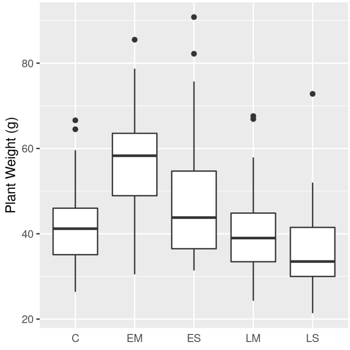

(**j**)

Figure S1. Phenotypic trait values across treatment groups.

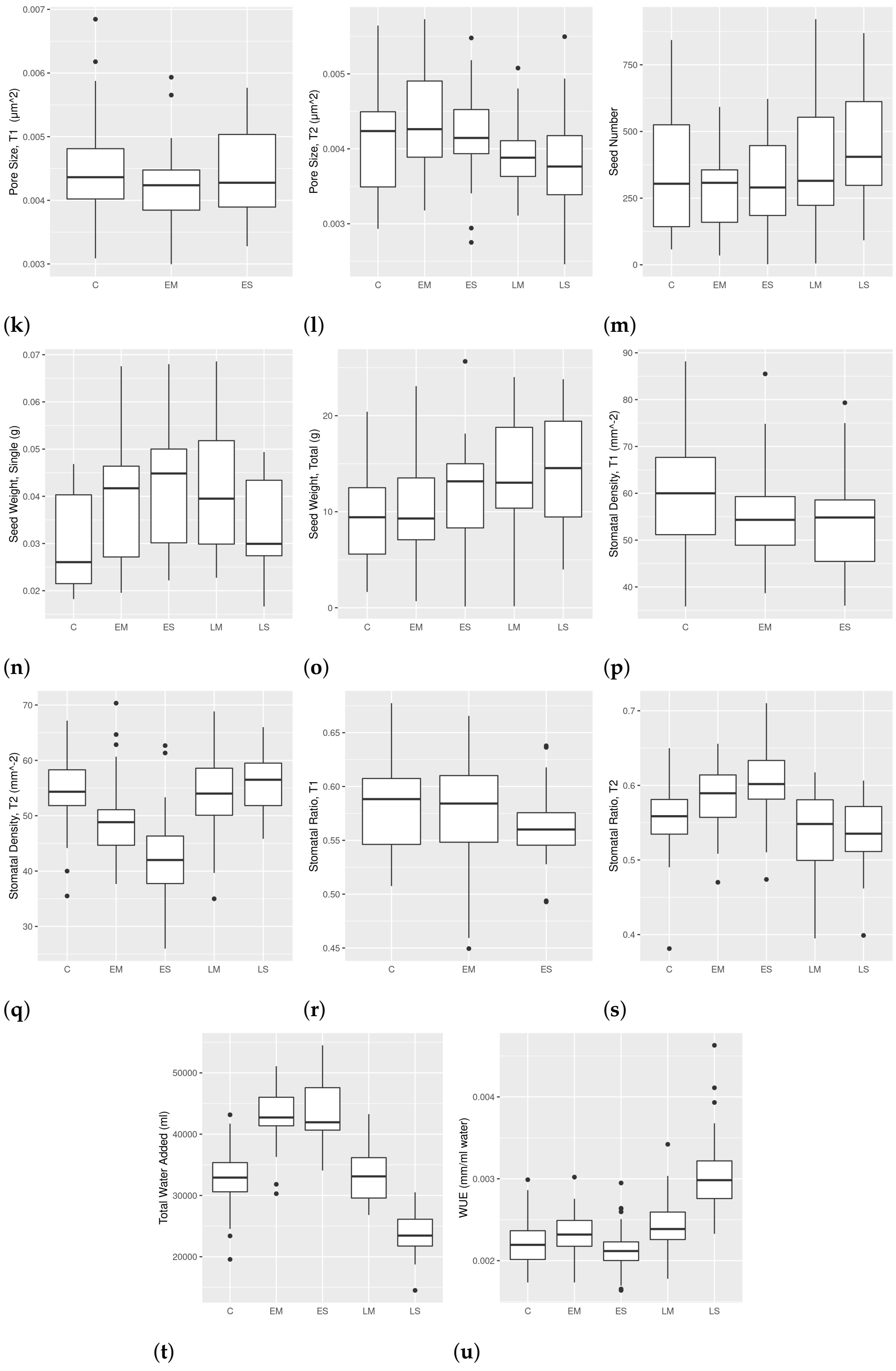

Figure S1. (Continued) Phenotypic trait values across treatment groups.

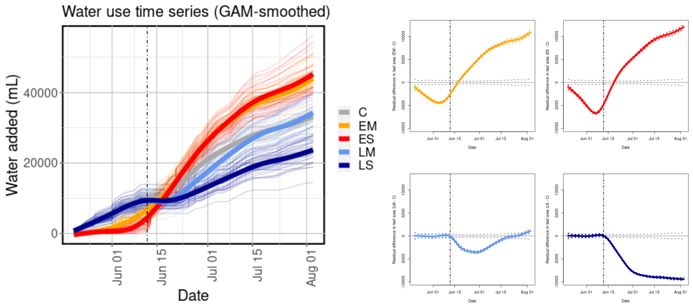

(**a**)

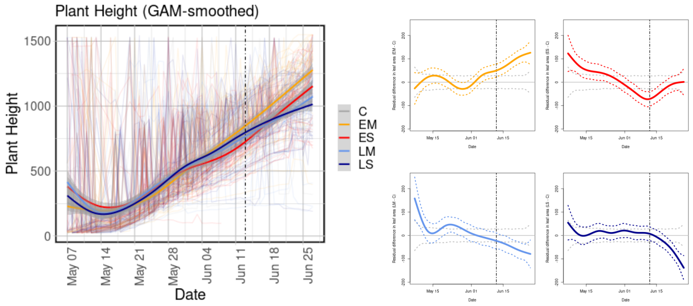

(**b**)

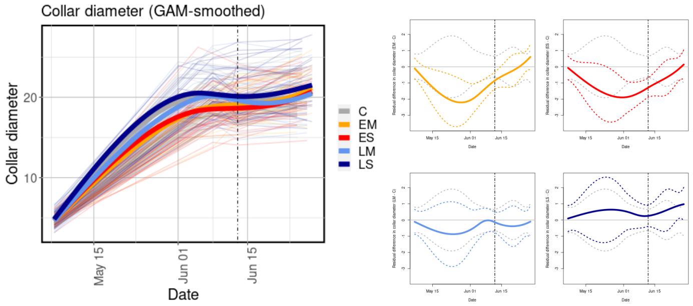

(**c**)

Figure S2. Time-series phenotypic trait values and residual plots. In the left panels, thin lines reflect individual plant data, and thick lines reflect generalized additive models. The right panels are residual plots of the four drought treatment group GAMs to the control group GAM. Dashed lines denote the 95% confidence intervals of the GAMs.

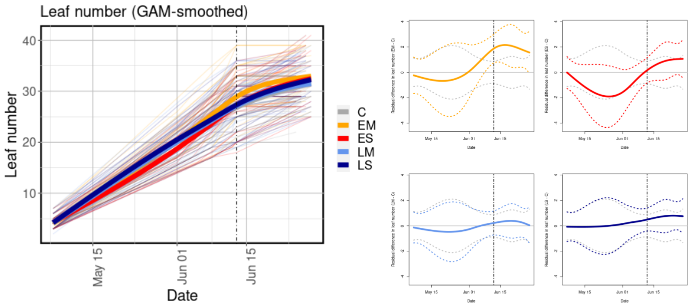

(**d**)

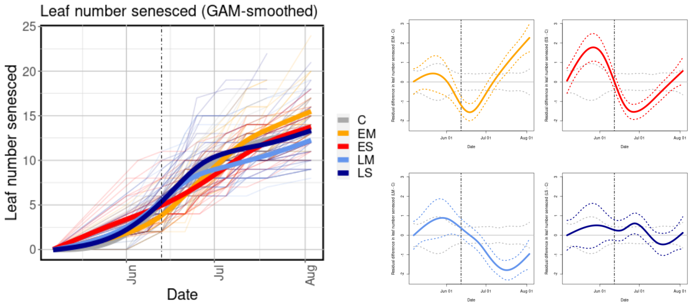

(**e**)

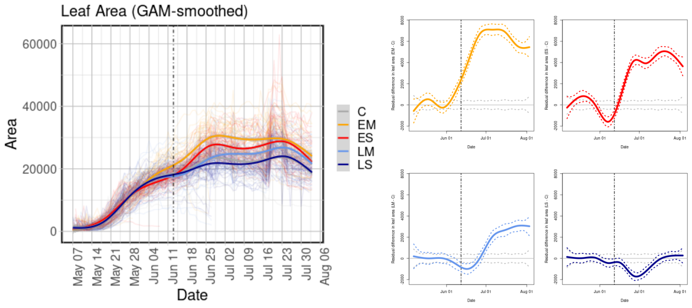

(**f**)

Figure S2. (Continued) Time-series phenotypic trait values and residual plots. In the left panels, thin lines reflect individual plant data, and thick lines reflect generalized additive models. The right panels are residual plots of the four drought treatment group GAMs to the control group GAM. Dashed lines denote the 95% confidence intervals of the GAMs.

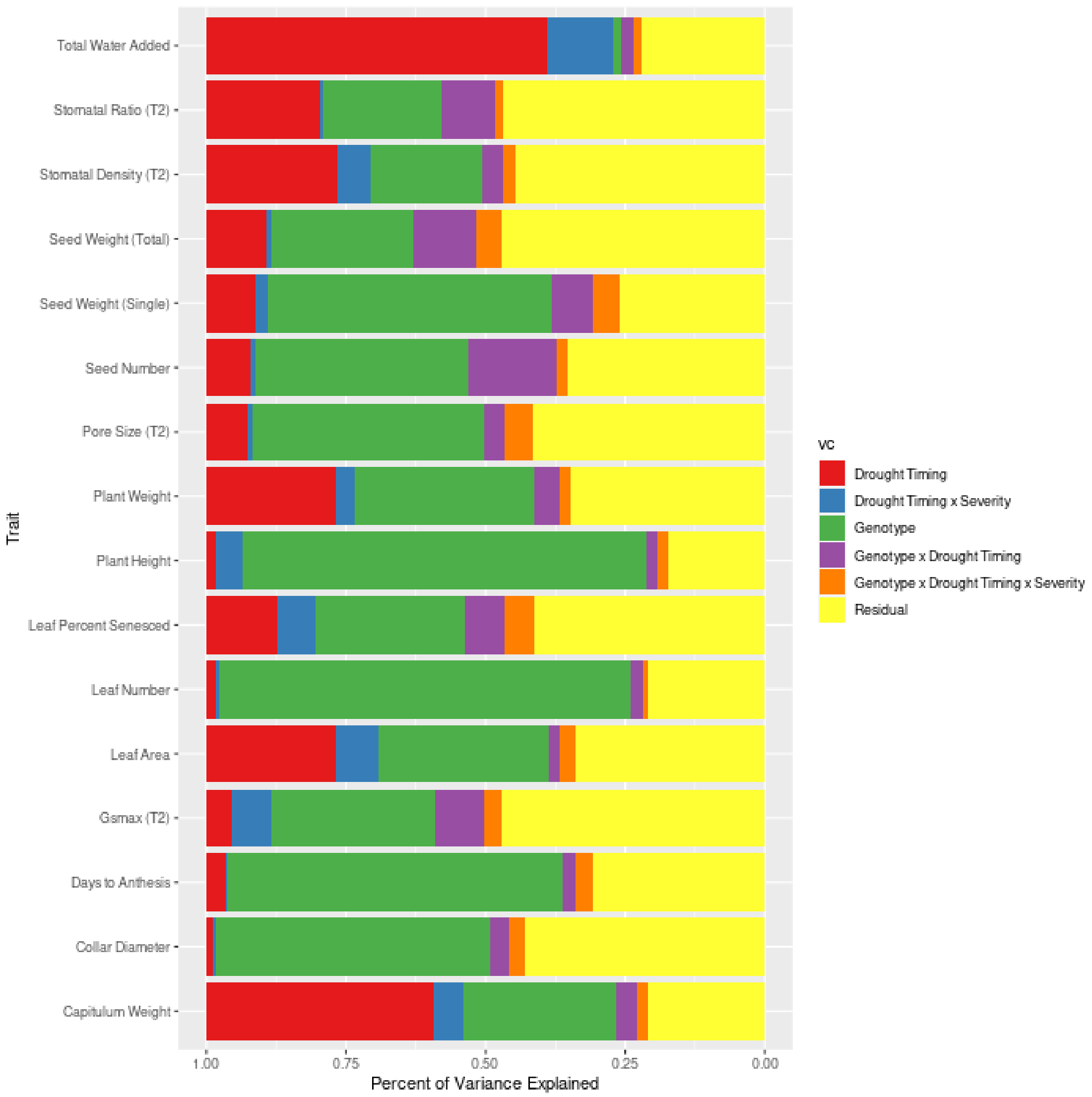

Figure S3. Percent of phenotypic variance explained by drought timing, drought severity, genotype, and interactions thereof.

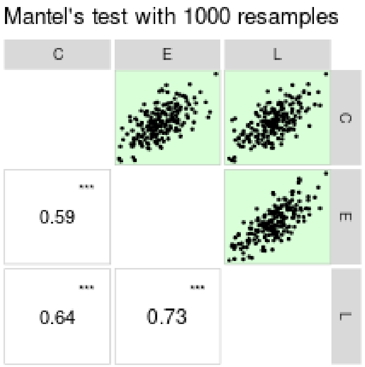

(**a**)

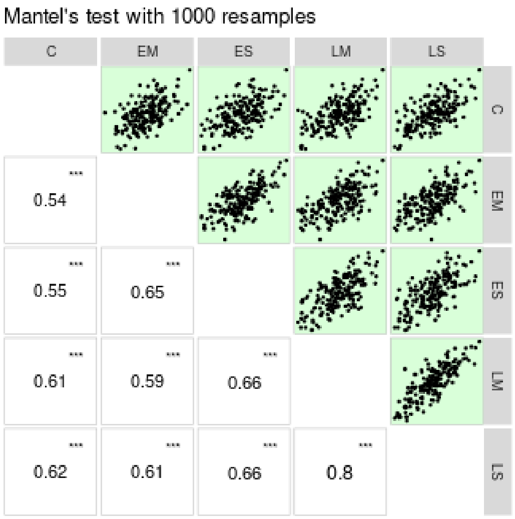

(**b**)

Figure S4. Mantel test of similarity between trait-trait Pearson correlation matrices. Larger values indicate more similar correlation matrices. All matrices are significantly similar (adjusted p-value $<$ 0.05). (a) Control, early, and late treatment group correlation matrices. (b) Control, EM, ES, LM, and LS treatment correlation matrices.

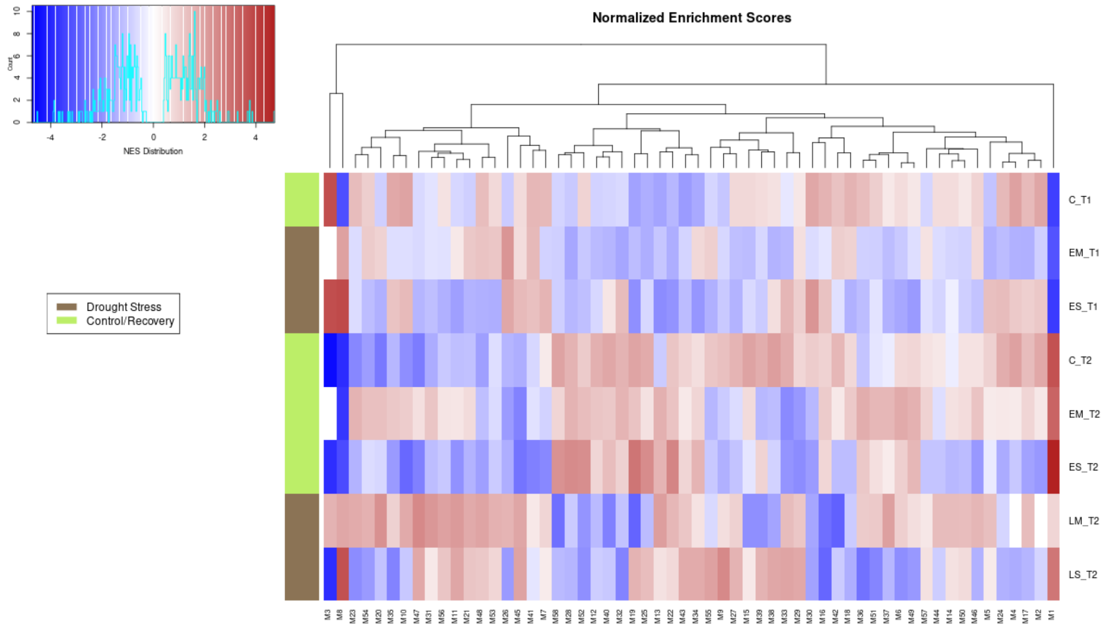

Figure S5. Normalized enrichment scores (NES) of all 58 gene modules. Modules are ordered by hierarchical clustering.

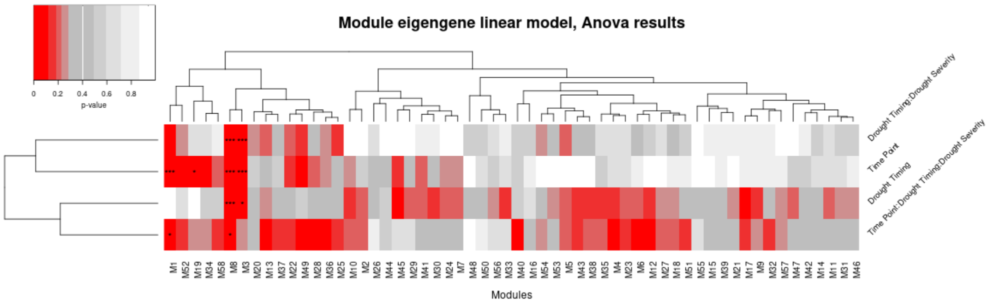

Figure S6. Significance of ANOVA main effects at explaining variance in gene module eigengene values. Significant effects are denoted with asterisks ($p < 0.05$, single asterisk; $p < 0.05/58 = 0.00086$, triple asterisks).

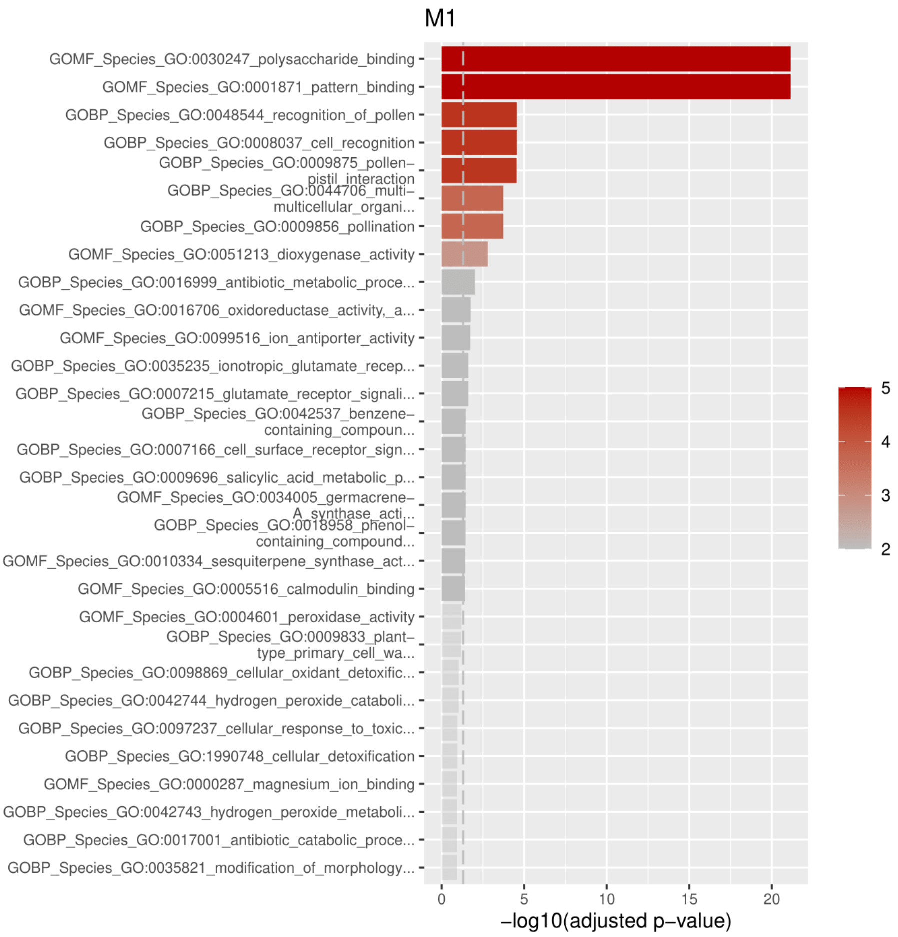

(**a**)

Figure S7. Overrepresentation Analysis of Modules M1 (a), M3 (b), M8 (c), M18 (d), M26 (e), and M52 (f).

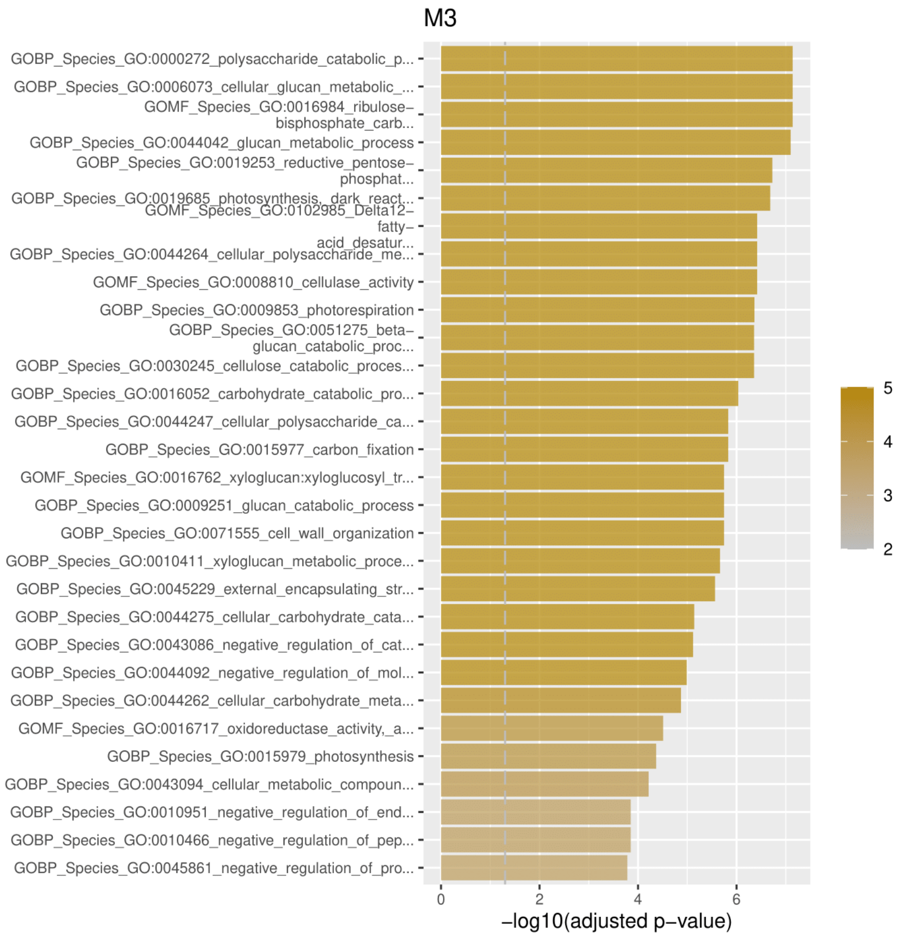

(**b**)

Figure S7. (Continued) Overrepresentation Analysis of Modules M1 (a), M3 (b), M8 (c), M18 (d), M26 (e), and M52 (f).

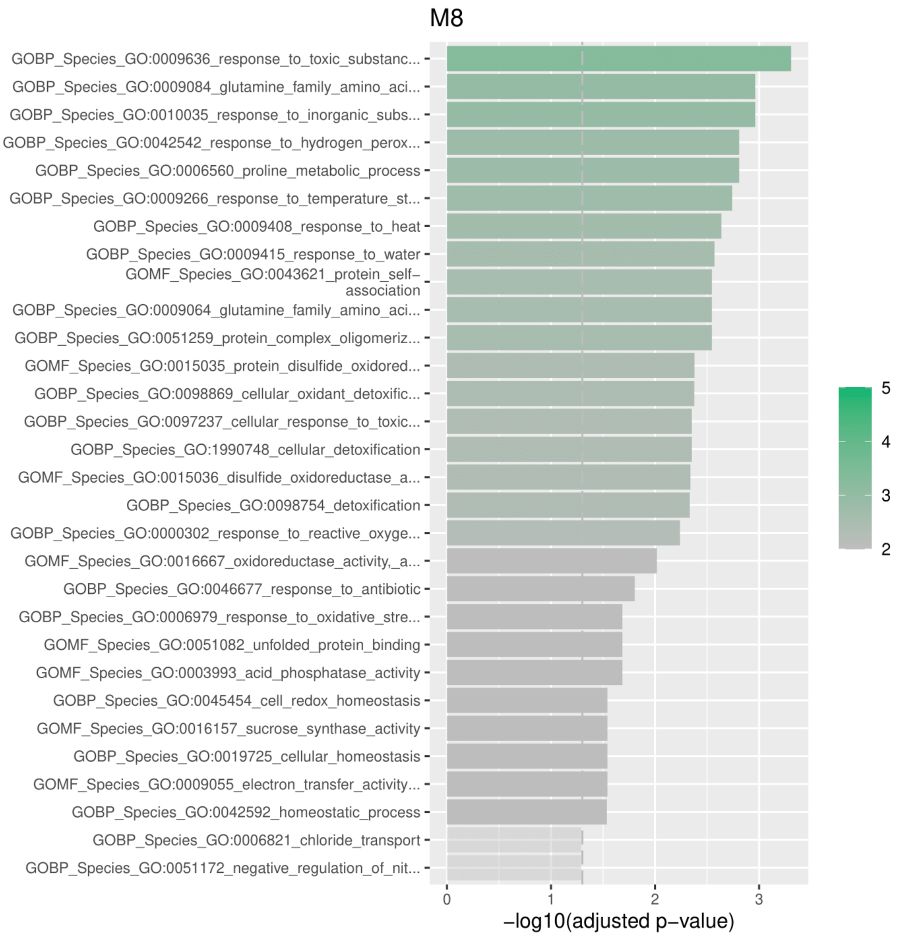

(**c**)

Figure S7. (Continued) Overrepresentation Analysis of Modules M1 (a), M3 (b), M8 (c), M18 (d), M26 (e), and M52 (f).

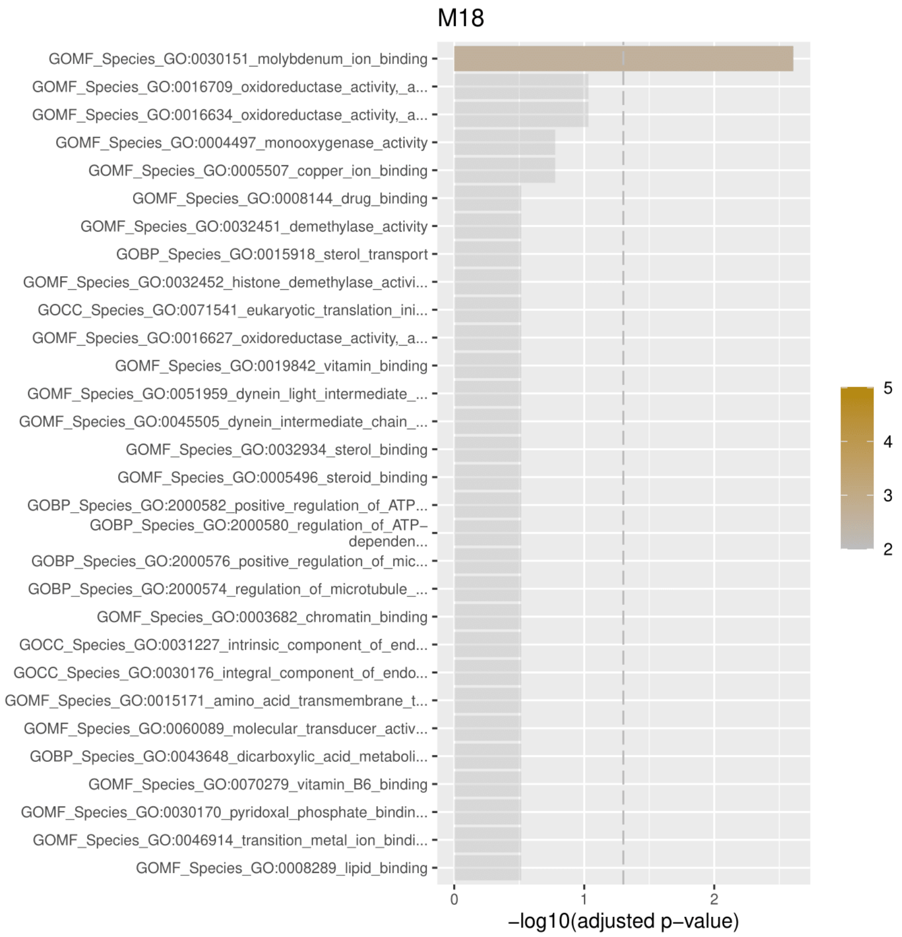

(**d**)

Figure S7. (Continued) Overrepresentation Analysis of Modules M1 (a), M3 (b), M8 (c), M18 (d), M26 (e), and M52 (f).

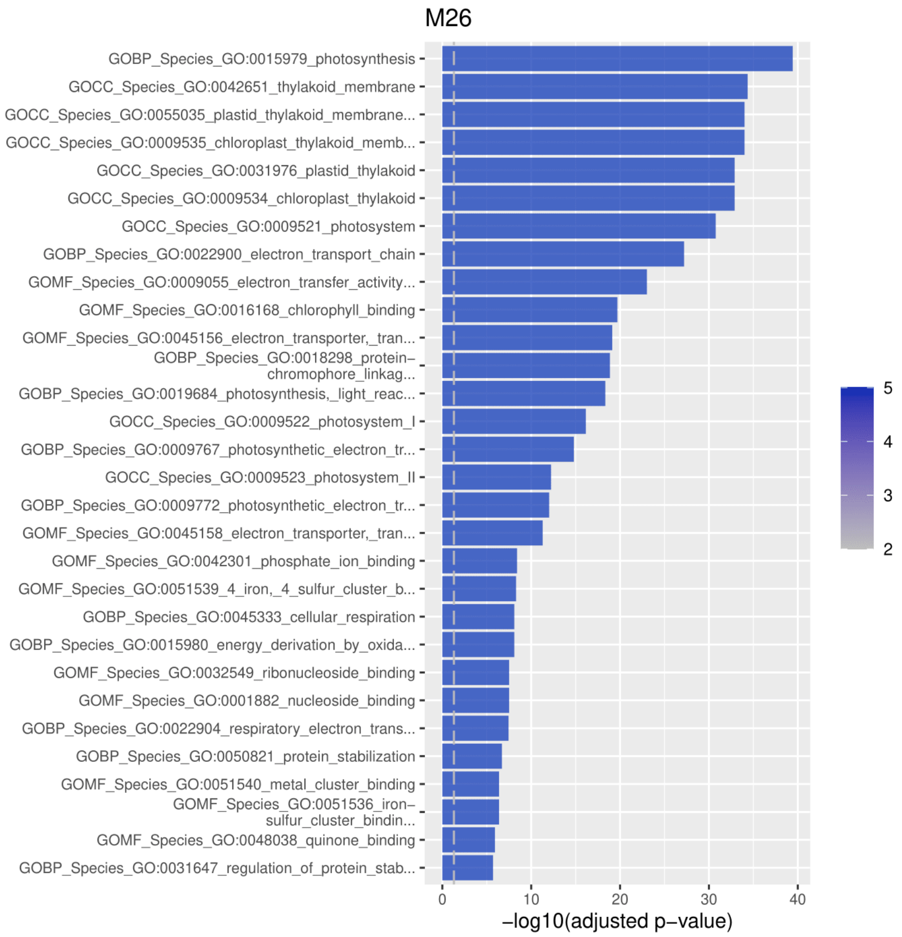

(**e**)

Figure S7. (Continued) Overrepresentation Analysis of Modules M1 (a), M3 (b), M8 (c), M18 (d), M26 (e), and M52 (f).

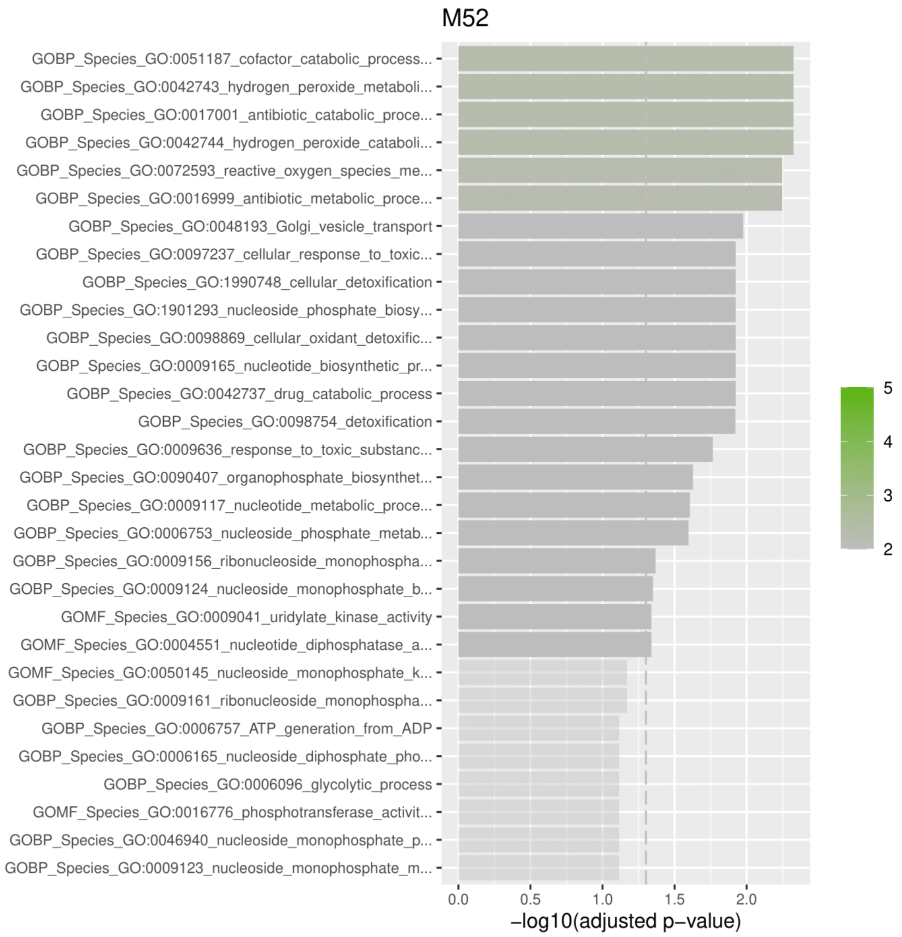

(**f**)

Figure S7. (Continued) Overrepresentation Analysis of Modules M1 (a), M3 (b), M8 (c), M18 (d), M26 (e), and M52 (f).

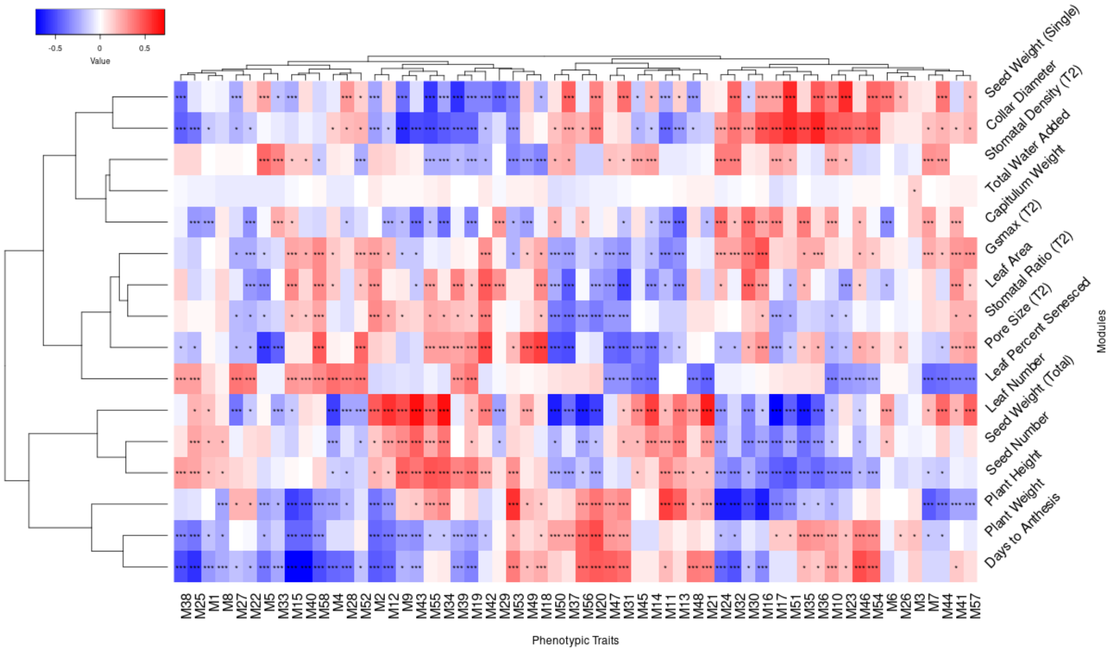

Figure S8. Pearson correlation between module eigengene and phenotypic trait values ($p < 0.05$, single asterisk; $p < 0.05/58 = 0.00086$, triple asterisks).

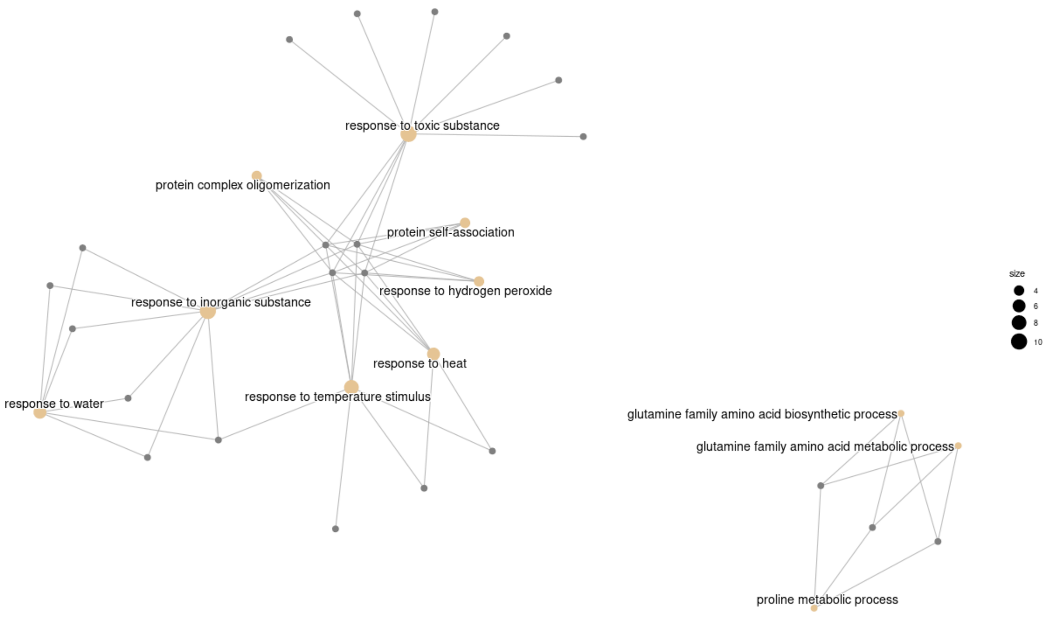

Figure S9. Gene-concept network plot of Module M8. Gray nodes represent genes and are connected by edges to orange nodes which represent enriched gene functions. Node sizes represent the number of genes in the module that are involved in a given function.

	C - E	C - L	E - L
Capitulum Weight	-12.5 ***	-3.9 ***	8.6 ***
Collar Diameter	0.841	0.2	-0.64
Days to Anthesis	0.865	0.053	-0.81
Gsmax (T1)	0.0851 ***	-	-
Gsmax (T2)	0.0623 ***	0.025	-0.037
Leaf Area	-5.66×10^4 ***	2.9×10^3	6×10^4 ***
Leaf Number	-1.48 ***	-0.53	0.95
Leaf Percent Senesced	-0.0421 ***	0.023	0.065 ***
Plant Height	-32.8	87 ***	1.2×10^2 ***
Plant Weight	-10.7 ***	5.1 *	16 ***
Pore Size (T1)	0.000268 *	-	-
Pore Size (T2)	-1.83×10^4	1.3×10^{-4}	3.1×10^{-4} ***
Seed Number	36.2	-87	-120 ***
Seed Weight (Single)	-0.00886 ***	-0.0066 ***	0.0022
Seed Weight (Total)	-1.68	-4.7 ***	-3 ***
Stomatal Density (T1)	4.12 *	-	-
Stomatal Density (T2)	8.2 ***	-1	-9.2 ***
Stomatal Ratio (T1)	0.0128	-	-
Stomatal Ratio (T2)	-0.0353 ***	0.017	0.052 ***
Total Water Added	-1.09×10^4 ***	4.3×10^3 ***	1.5×10^4 ***
Water Use Efficiency	8.06×10^{-6}	-5.20×10^{-4} ***	-5.29×10^{-4} ***

Table S1. Estimated marginal means of phenotypic traits for drought treatment groups Control (C), Early drought stress (E), and Late drought stress (L). Significant differences in trait means are denoted with asterisks ($p < 0.05$, single asterisk; $p < 0.05/3 = 0.0167$, triple asterisks).

	C - EM	C - ES	C - LM	C - LS	EM - ES	LM - LS
Capitulum Weight	-12.3 ***	-13 ***	-6.99 ***	-0.83	-0.456	6.2 ***
Collar Diameter	0.678	1	0.458	-0.064	0.327	-0.52
Days to Anthesis	0.681	1	0.244	-0.14	0.369	-0.38
Gsmax (T1)	0.0874 ***	0.083 ***	-	-	-0.00447	-
Gsmax (T2)	0.0202	0.1 ***	0.0261	0.024	0.0844 ***	-0.0016
Leaf Area	-8.51×10^4 ***	-2.8×10^4	2.17×10^3	3.7×10^3	5.7×10^4 ***	1.6×10^3
Leaf Number	-1.89 *	-1.1	-0.128	-0.93	0.813	-0.8
Leaf Percent Senesced	-0.0702 ***	-0.014	0.0445	0.0016	0.0561 *	-0.043
Plant Height	-115 ***	49	78.8	96 *	164 ***	17
Plant Weight	-14.4 ***	-7 *	3.63	6.7	7.45 *	3
Pore Size (T1)	0.000276	0.00026	-	-	-1.57×10^{-5}	-
Pore Size (T2)	-0.000257	-0.00011	6.22×10^{-5}	0.00019	0.000148	0.00013
Seed Number	43.5	29	-55.8	-1.2×10^2	-14.6	-63
Seed Weight (Single)	-0.00723 ***	-0.01 ***	-0.00913 ***	-0.0042	-0.00325	0.005
Seed Weight (Total)	-0.986	-2.4	-4.14 *	-5.2 ***	-1.38	-1.1
Stomatal Density (T1)	4.69	3.5	-	-	-1.15	-
Stomatal Density (T2)	4.99 *	11 ***	-0.0451	-2	6.43 ***	-1.9
Stomatal Ratio (T1)	0.00548	0.02	-	-	0.0146	-
Stomatal Ratio (T2)	-0.0285	-0.042 ***	0.0148	0.019	-0.0135	0.0039
Total Water Added	-1.03×10^4 ***	-1.1×10^4 ***	-548	9.2×10^3 ***	-1.1×10^3	9.7×10^3 ***
Water Use Efficiency	-6.03×10^{-5}	7.64×10^{-5}	-1.85×10^{-4}	-8.56×10^{-4} ***	1.37×10^{-4}	-6.71×10^{-4} ***

Table S2. Estimated marginal means of phenotypic traits for drought treatment groups control (C), early moderate stress (EM), early severe stress (ES), late moderate stress (LM), and late severe stress (LS). Significant differences in trait means are denoted with asterisks ($p < 0.05$, single asterisk; $p < 0.05/6 = 0.0083$, triple asterisks).

T1

Category	All Genes	DEGs
$log2fc_{EM-C} > log2fc_{ES-C}$	14,480	343
$log2fc_{EM-C} < log2fc_{ES-C}$	20,431	843
Divergent	21,355	3
p-value	$4.747634 \times 10^{-113}$	1.315256×10^{-25}

T2

Category	All Genes	DEGs
$log2fc_{LM-C} > log2fc_{LS-C}$	19,986	627
$log2fc_{LM-C} < log2fc_{LS-C}$	22,955	472
Divergent	13,325	0
p-value	4.050325×10^{-24}	1.06941×10^{-3}

Table S3. Counts of genes that are more differentially expressed (i.e., have greater absolute value of $\log_2$ fold change, *log2fc*) between control and either moderate or severe stress. *P*-values reflect two-proportion z-tests between stress groups of differing severity and denote the probability that the two proportions are not significantly different. Divergent genes (i.e., genes with *log2fc* values of opposite sign) are not included in the statistical comparison of proportions. Tests are distinguished between stress at T1 and T2, and between considerations of all genes or of the subset of DEGs in both stress groups relative to the concurrent control.

T1

Statistic	All Genes	DEGs
Mean Difference ($log2fc_{EM-C} - log2fc_{ES-C}$)	-0.01030038	-0.1851118
t	-4.1987	-14.122
df	56,265	1,188
p-value	2.689×10^{-5}	5.617264×10^{-42}

T2

Statistic	All Genes	DEGs
Mean Difference ($log2fc_{LM-C} - log2fc_{LS-C}$)	-0.06333261	0.05426942
t	-22.261	5.2029
df	56,265	1,098
p-value	$2.633496 \times 10^{-109}$	2.340126×10^{-7}

Table S4. Statistical summary of paired sample t-tests of per-gene difference in $\log_2$ fold change (*log2fc*) in the moderate stress-control contrast and the severe stress-control contrast. Divergent genes (i.e., genes with *log2fc* values of opposite sign) are not included in the statistical comparison of magnitudes of DE. Negative mean difference values mean that the severe stress group had greater absolute values of *log2fc* than did the moderate stress group. P-values denote the probability that the mean difference in magnitude of *log2fc* between these groups is 0 (the alternative hypothesis is two-tailed). Tests are distinguished between stress at T1 and stress at T2, and between considerations of all genes or the subset of DEGs in both stress groups relative to the concurrent control.
